# Synthesis and Application of Zero-Valent Iron Nanoparticles in Water Treatment, Environmental Remediation, Catalysis, and Their Biological Effects

**DOI:** 10.3390/nano10050917

**Published:** 2020-05-09

**Authors:** Tibor Pasinszki, Melinda Krebsz

**Affiliations:** Department of Chemistry, School of Pure Sciences, College of Engineering, Science and Technology, Fiji National University, Suva P.O. Box 7222, Fiji; melinda.krebsz@gmail.com

**Keywords:** nanoiron, nZVI, organic pollutant removal, heavy metal removal, soil remediation, wastewater treatment, nanotoxicity

## Abstract

Present and past anthropogenic pollution of the hydrosphere and lithosphere is a growing concern around the world for sustainable development and human health. Current industrial activity, abandoned contaminated plants and mining sites, and even everyday life is a pollution source for our environment. There is therefore a crucial need to clean industrial and municipal effluents and remediate contaminated soil and groundwater. Nanosized zero-valent iron (nZVI) is an emerging material in these fields due to its high reactivity and expected low impact on the environment due to iron’s high abundance in the earth crust. Currently, there is an intensive research to test the effectiveness of nZVI in contaminant removal processes from water and soil and to modify properties of this material in order to fulfill specific application requirements. The number of laboratory tests, field applications, and investigations for the environmental impact are strongly increasing. The aim of the present review is to provide an overview of the current knowledge about the catalytic activity, reactivity and efficiency of nZVI in removing toxic organic and inorganic materials from water, wastewater, and soil and groundwater, as well as its toxic effect for microorganisms and plants.

## 1. Introduction

**Figure 1:**
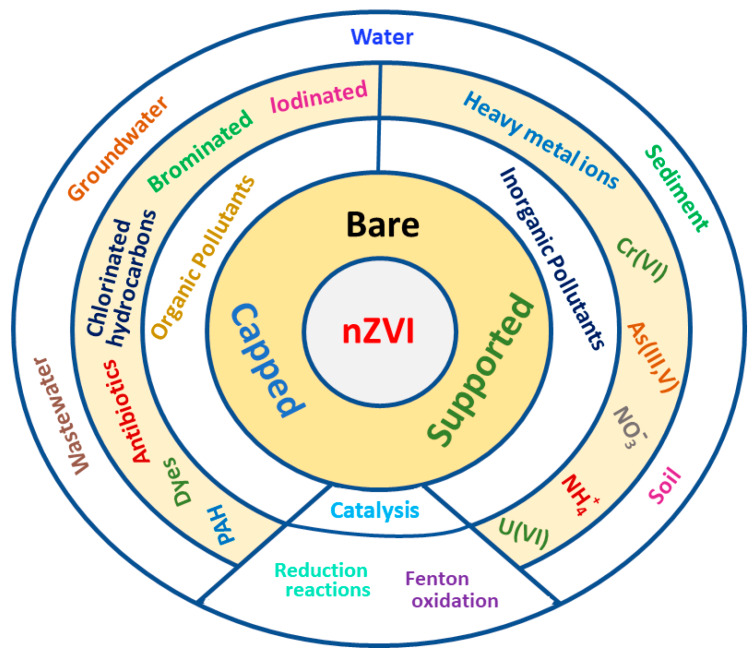
Application fields of nanosized zero-valent iron (nZVI) in water purification and environmental remediation.

Iron is the fourth most abundant element in the earth crust and its application in human history goes back about 3200 years, to the beginning of the Iron Age. Despite of this long history, the 20th century brought new application fields and challenges for metallic iron due to the emerging nanotechnology and with the production of nanosized zero-valent iron, nZVI. nZVI has markedly different properties compared to bulk iron, and it has now become one of the most important engineered nanomaterials, and the most common nanomaterial for environmental remediation due to its relatively low cost, high reactivity, and good adsorption capacity [1,2,3,4]. nZVI has strong reducing power and it is sufficiently reactive toward a large number of organic and inorganic compounds, including halogenated hydrocarbons, organic dyes, antibiotics, heavy metal ions, etc. nZVI reacts rapidly with oxygen and water, and has a high tendency to agglomerate; therefore, these nanoparticles, NPs, are usually capped with various inorganic and organic materials or supported on various supports. Capping and support agents consequently reduce the reactivity in some extent, what is gained by the small size of NPs, but increase stability and transport properties, what is essential for environmental, e.g., soil and groundwater, remediation. The potential of nZVI for environmental remediation has initiated numerous laboratory works for investigating the reactivity and toxicity of these NPs with distinct inorganic and organic contaminants and microorganisms and plants, respectively, as well as field applications, especially during the last two decades [1,5,6,7]. Currently, application studies of nZVI are strongly increasing, and the aim of the present work is to summarize developments on this lucrative field. Figure 1 summarizes major application fields of nZVI and the target of the present review.

## 2. Synthesis of Zero-Valent Iron Nanoparticles

Properties of nZVI, such as reactivity and mobility, strongly depend on its size, surface, modifying capping material, oxide layer, or support material, consequently on its manufacturing process. Application requirements of nZVI also determine the method of choice of synthesis. Bare nZVI is very reactive, estimated to be 10–1000 times more reactive than granular ZVI [8], and agglomerates due to its high surface energy and magnetic properties. When in contact with air and water, nZVI is always covered with a thin oxide layer. In water, this layer appears to be a mixed Fe(0)/Fe(II)/Fe(III) phase with an apparent surface stoichiometry close to FeOOH [9]. The major phase is lepidocrocite (γ-FeOOH) [10]. A core–shell iron particle with the proper thickness of the oxide shell, which does not block electron transfer from the iron core can be more advantageous in practical applications due to higher stability than bare pyrophoric iron NPs. Agglomeration of nZVI can be prevented by supporting and dispersing NPs on solid materials. The colloidal properties can be improved by modifying the surface using organic polymers and polyelectrolytes. Capping or supporting NPs can be done simultaneously or in a consecutive step of their synthesis. nZVI, in general, can be produced using physical or chemical methods either by reducing the size of bulk iron to nanoscale (top-down approach) or building up nanoiron from atoms generated from ions or molecules (bottom-up approach) [8,11].

### 2.1. Top-Down Synthesis

**Figure 2:**
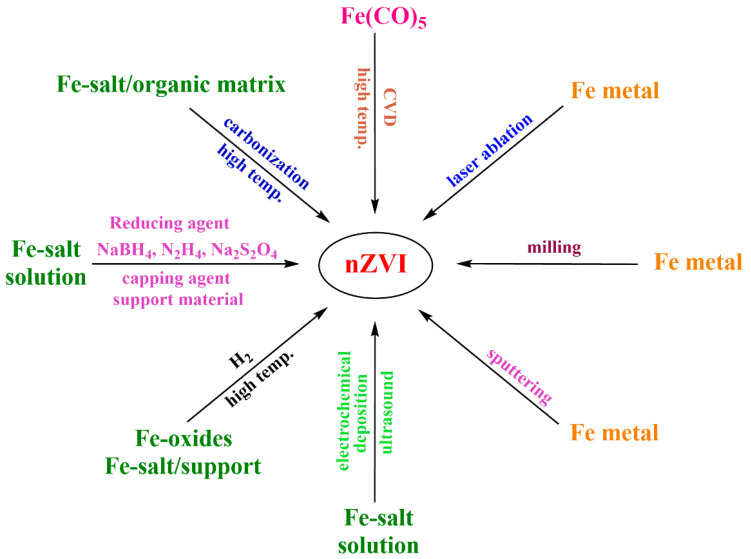
Top-down (processing micro- or millimeter-sized Fe metal) and bottom-up (using Fe-salts or Fe-compounds as starting materials) synthetic methods for the production of nZVI. The most frequently used reagents and reaction conditions are shown in the Figure.

Top-down methods for nZVI production are the milling processes, pulsed laser ablation, and noble gas sputtering (Figure 2). 

The most frequently used top-down method is the milling process. It starts from micrometer- to millimeter-sized iron filings (e.g., carbonyl iron, sponge iron, cast iron, and iron powder), which are milled (using ball-mills, vibrating mills, and stirred ball mills) to fine, nanosized particles. One of the advantages of this method is that it is easy to scale-up. In fact, this method is used in the industry to produce nZVI on a large scale. Another advantage is that the method does not require the use of expensive and toxic chemicals (compare, e.g., to the borohydride solution method below). The milling process determines the size and shape of final NPs, which has a huge impact on particle reactivity. Milling in an inert atmosphere produces very reactive and pyrophoric iron particles, which ignite spontaneously upon contact with air. To reduce combustion hazard and reactivity of NPs, a capping layer is usually developed during the process by performing milling in a grinding media. If the media contains water, a stabilizing oxide shell forms on NPs, but the side product hydrogen creates a combustion hazard. To solve stability and safety issues, several grinding media and additives, as well as grinding equipment, the iron source, and milling times were tested.

Li et al. developed a solvent-free production of nZVI using precision ball milling, comprising of stainless steel beads in a high-speed rotary chamber, and micro iron powder as the starting material [12]. The micro iron was effectively reduced to irregular flaky shape particles with sizes between 10 and 50 nm after 8 h of milling. nZVI prepared by this process contained magnetite and maghemite surface oxides. Ribas et al. constructed a two-step wet milling process using alumina as an abrasion agent to reduce NP size [13]. In the first step, using conventional ball milling and monoethylene glycol (MEG) solvent, carbonyl iron particles were reduced to very thin flakes. Alumina was introduced in the second step, and the flakes were broken to smaller iron particles. No evidence of thick and continuous oxide layer on the surface was observed. Köber et al. developed a two-stage top-down process, where the first step involved dry grinding with corrosion inhibitors to micrometer size and the second step involved grinding in liquid and addition of a surfactant [14]. Gao et al. ball milled microsize iron and activated carbon powders using zirconia beads and propyleneglycol as lubricant [15]. Iron NPs were dispersed in activated carbon in the product.

Noble gas sputtering is a less frequently used method for nZVI synthesis and mainly used in specialized laboratories. If iron is used as the sputtering target, a supersaturated iron vapor forms in the sputtering process, which can be condensed into solid clusters by cooling. Kuhn et al. prepared core–shell iron–iron oxide (γ-Fe_2_O_3_/Fe_3_O_4_) nanoparticles by using an iron hollow cylinder as the cathode and argon as the sputtering gas in a high-vacuum chamber, and oxidizing iron NPs in a consecutive step by exposing them to an increasing pressure of oxygen [16].

Laser ablation is another method to synthesize Fe NPs on a small scale. Irradiation of an iron metal target with a laser pulse locally melts and vaporizes the metal. Hot metal atoms are cooled by the surrounding medium and form metal NPs. If the solution contains oxygen or water, NPs easily oxidized. Okazoe et al. developed a method to produce nZVI NPs with a mean size of 7.1 nm by laser ablation under atmospheric conditions using the reducing properties of formate-based ionic liquid solvents, namely tetraoctylphosphonium formate and tetraoctylphosphonium bis(trifluoromethyl-sulfonyl)imide [17].

### 2.2. Bottom-Up Synthesis

Bottom-up approaches to build nZVI start from either dissolved iron salts, nanosized iron oxides, or iron-containing molecules (Figure 2). 

#### 2.2.1. Solution Based Processes

The most frequently used method for nZVI synthesis in laboratories is the borohydride route, where iron salts, typically chlorides, are reduced in aqueous environment under inert atmosphere with sodium borohydride (Equations (1) and (2)). There is an economically unfavorable side reaction in this synthesis, namely the borohydride hydrolysis (Equation (3)). Due to this latter, the synthesis of nZVI requires the application of large excess of this toxic and expensive reagent. The typical diameter of NPs obtained in this process is below 100 nm. The reactivity of NPs strongly depends on the pH of the solution, and the reactivity is higher if the precursor concentration and the feeding rate of borohydride solution are higher. Barreto-Rodrigues et al., for example, obtained the highest reactivity for nZVI when a high rate of addition of NaBH_4_, low pH (2–3), and a Fe^2+^:BH_4_^−^ molar ratio of 1:3 were used during the synthesis. The product contained NPs with a mean crystallite diameter of 70 nm and sphere-like morphology in chain aggregates [18]. Han et al. optimized the synthesis conditions of nZVI and observed that the reductive reactivity of nZVI was most sensitive to the initial concentration of the iron precursor, borohydride feed rate, and the loading ratio of borohydride to ferric ion. Solution mixing speed, however, had little influence [19]. Ultrasound irradiation during the synthesis was shown to influence strongly the particle size and shape; under high ultrasonic power the spherical morphology of NPs changed to the plate and needle type. The particle size also decreased when high ultrasonic power and high precursor/reductant ratio were used [20]. Kamali et al. studied the effect of the BH_4_^−^/Fe^3+^ ratio, the Fe^3+^ concentration, reductant addition rate, and ultrasonic irradiation on the crystallinity and specific surface area of nZVI particles [21]. It was observed that both the BH_4_^−^/Fe^3+^ ratio and the Fe^3+^ concentration determined the particle crystalline phase composition, crystallinity, and surface area; the ratio, however, was revealed to be the most important. The effect of the reductant addition rate was negligible. Ultrasonic irradiation resulted in smaller particles, therefore in higher surface area.

2 Fe^2+^ + BH_4_^−^ + 3 OH^−^ → 2 Fe^0^ + H_3_BO_3_ + 2 H_2_ (1)

4 Fe^3+^ + 3 BH_4_^−^ + 9 OH^−^ → 4 Fe^0^ + 3 H_3_BO_3_ + 12 H_2_ (2)

BH_4_^−^ + 4 H_2_O → H_3_BO_3_ + OH^−^ + 4 H_2_ (3)

The chemical reduction method is complex and includes multiple steps such as preparation of the supersaturated solution, nucleation of the nZVI cluster, growth of nZVI nuclei, and agglomeration of nZVI, as well as washing, separation, and dehydration of NPs. These latter steps may lead to the formation of thin capping oxide layer on the iron surface depending on conditions [22]. The borohydride route is widely used also for synthesizing capped and supported nZVI (see below), where this reaction is performed at the presence of a capping material or solid support. Other much less frequently used solution methods are based on the application of hydrazine [23] or sodium dithionite [24] as the reducing agent instead of NaBH_4_, or on the decomposition of iron pentacarbonyl in a high boiling point solvent [25].

To minimize aggregation, increase stability, increase mobility, and reduce leaching of nZVI, a wide range of inorganic and organic support and capping materials are used [8]. Inorganic materials used to date include aluminum hydroxide [26], pumice [27], Mg-aminoclay [28], SBA-15 silica [29], kaolin, montmorillonite, bentonite, zeolite, sepiolite, marine clay, coral, oyster shell, biochar, carbon black, cation exchange membrane, calcium-alginate bead, silica fume, graphene oxide (GO), and multiwalled carbon nanotubes (MWCNTs) [8]. The synthesis of nZVI/inorganic carrier materials is usually based on the chemical process using NaBH_4_ aqueous solution, where carriers are added to the iron ion solution before NaBH_4_. Organic capping and support materials can be monomers, surfactants, or polymers, and may provide hydrophobic, hydrophilic, or amphiphilic properties for nZVI organic composites. Examples for organic modifiers are pectin [30], long-chain carboxylic acids and long-chain amines [25], rhamnolipid [31], octa(cholinium)-polyhedral oligomeric silsesquioxane [32], sycamore tree seed pod fibers [33], polyvinyl-pyrrolidone (PVP), polyethylene glycol (PEG), carboxymethyl cellulose (CMC), polyacrylic acid (PAA), polystyrene resin, and polyvinyl alcohol-co-vinyl acetate-co-itaconic acid [8]. All of these organic modifier/nZVI composites can be prepared by the borohydride route. The sizes of NPs depend on experimental conditions and the amount and kind of organic materials.

The borohydride synthetic route is very popular in laboratories for producing nZVI on a small-scale due to its simplicity. Scaling-up the process is a requirement for large-scale applications. Vilardi et al. developed recently an intensified production of nZVI using a spinning disk reactor [34]. Highly uniform particles with a low mean size of 28 nm were obtained and the lab-scale equipment could produce nZVI in the range of 0.24–24 kg/day depending on the initial Fe(II) concentration. Scaling-up processes were also optimized for nZVI production in laboratory-scale stirred tank reactors [35,36]. Jiao et al. [37] and Fan et al. [38] developed continuous processes for nZVI production using impinging stream-rotating packed bed reactors. Using this technique, the preparation time was shorter, the particle size was smaller, and the particle size distribution was narrower compared to those of stirred tank reactors [37,38]. nZVI particles obtained had quasispherical morphology and almost uniformly distributed with a particle size of 10–20 nm [37]. Wang et al. also developed a process where nZVI particles were continuously prepared using a rotating packed bed reactor with stainless wire mesh packing [39]. Particles with an average size of 14 nm were obtained. Compared to a stirred tank reactor, the rotating packed bed reactor produced particles with a smaller particle size and a much shorter reaction time.

Green methods for producing nZVI are becoming more important due to safety and environmental concerns [40]. In this respect the replacement of borohydride with green alternatives is the key issue. The green production method uses extracts from natural products such as tree leaves, fruits, etc. having high antioxidant capacities, therefore components that react with iron ions in solution to produce nZVI particles. The advantages of this method are the use of non-toxic reducing agent, the natural capping of the NPs by the extract matrix, and valorization of natural products. Due to capping they have less tendency to agglomerate and have prolonged reactivity. A wide range of natural products or their extract have been used to date such as oak leaves extract [41], apple, apricot, avocado, cherry, eucalyptus, kiwi, lemon, mandarin, medlar, mulberry, oak, olive, orange, passion fruit, peach, pear, pine, pomegranate, plum, quince, raspberry, strawberry, tea-black, tea-green, vine, and walnut leaf extracts [42], wastes from citrine juice (orange, lime, lemon, and mandarin) [43], and yeast extract [44].

Electrochemical deposition of iron from solutions is a low-cost and high-efficient method to synthesize ZVI. Material particles synthesized this way are usually larger than the nanometer domain, for example, the three-dimensional hierarchical dendritic ZVI with nano-dimensions only in one dimension [45]. However, by combining electrochemical and ultrasonic techniques thereby removing nZVI NPs instantaneously from the cathode into the solution and using cetylpyridinium chloride surfactant to stabilize NPs, Chen et al. could produce NPs with diameter of 1–20 nm [46].

#### 2.2.2. High-Temperature Thermal Processes

When the support material is heat resistant or there are minor changes in its structure upon heating, the high-temperature processes is based on soaking the support with iron salts, drying, and reducing iron salts on the support surface above 500 °C using gaseous reducing agents directly, e.g., as hydrogen gas atmosphere [47,48] or H_2_/Ar plasma [49]. If graphene oxide (GO) is used as the support, it is reduced to reduced graphene oxide (rGO) under these conditions [47,49].

There are two common strategies for the preparation of composites of carbon materials and nZVI. One is the solution based method discussed above where preobtained carbon materials are decorated with metal salts or oxides, and then reduced by chemical methods. The other is the direct pyrolysis of prepared mixtures of organic materials and iron salts or oxides under an inert atmosphere; the high temperature (typically above 600 °C) applied in this process can induce the carbonization of organic materials, and iron salts or oxides are converted into iron NPs by synchronous carbon-thermic reduction. The synthesized carbon acts as the supporting matrix for the production and dispersion of nZVI. A wide range of materials has been used for this production to date, including metal‒organic frameworks (MOF) [50], ion exchange resins [51], alginate [52], and magnetite nanoparticles coated with lignin-derived biobased substances isolated from composted urban biowastes [53]. The phase purity of iron depends on the temperature of carbonization as very high temperature favors the formation of cementite, Fe_3_C [51].

nZVI is produced on industrial scale from nanogoethite or nanohematite, prepared from iron scrap by dissolving in diluted sulfuric acid and precipitation by pH adjustment, by treating these nano-oxides in hydrogen atmosphere at elevated temperatures [11,54]. The reduction process produces pyrophoric NPs, which are cooled and quenched with deionized water in consecutive steps. Freshly produced material has a Fe_3_O_4_ shell/iron core core–shell structure and a particle size in the range of 50–300 nm.

The chemical vapor deposition (CVD) technique, by pyrolyzing thermally labile organoiron compounds in the gas-phase, provides a route to nZVI. Iron pentacarbonyl is typically used in this process, however, the toxicity of this compound is a major drawback for its application. When Fe(CO)_5_ was heated in an inert gas stream above 350 °C pyrophoric iron NPs were produced with mean size between 5 and 13 nm, and with a core–shell structure [55]. Increasing decomposition temperature favored the formation of larger particles.

Visentin et al. analyzed the environmental and economic impacts of three industrial nZVI production methods used in the remediation of contaminated sites, namely the milling, the sodium borohydride route, and the chemical reduction with hydrogen gas [56]. Based on the evaluation of lifecycle costs and environmental impacts, the borohydride route was found to have the best environmental performance and the milling method was found to have the best economic performance. nZVI production based on reduction with hydrogen gas was the worst considering both environmental and economic impacts.

## 3. Application of nZVI in Catalysis

Iron-based catalysts attract much attention recently as potential greener alternatives of heavy metals, as well as due to their non-toxic nature and easy recyclability using an external magnetic field. The application of bare nZVI in a catalytic system, however, is challenging due to its poor chemical stability. nZVI is easily passivated on its surface in air or an aqueous environment and prone to aggregation, where both appear as the main drawbacks for catalytic applications. A possible strategy to avoid oxidation of nZVI is to trap NPs inside a suitable solid matrix without destroying the accessibility of catalytic sites. Goswami et al. have developed a nZVI/reduced graphene oxide (rGO) composite by thermal treatment of ferric nitrate-soaked GO under hydrogen atmosphere and successfully applied it for the catalytic reduction of aromatic nitro compounds to amines using hydrazine as the reagent [47] (Table 1). rGO protected nZVI from aerial oxidation and provided superior charge transfer to substrate molecules. nZVI NPs catalyze the reduction of *p*-nitrophenol to *p*-aminophenol using NaBH_4_ [57]. It has been shown that applying the nZVI/NaBH_4_ system in this reduction is advantageous because NaBH_4_ disintegrates nZVI NPs due to chemical etching the outermost oxide layer, resulting in smaller particles and increased reactive surface for p-nitrophenol reduction, and protects them from oxidation; this leads to good recyclability of the nZVI catalyst.

Capping nZVI increases its stability. Parimala and Santhanalakshmi synthesized polyvinyl-pyrrolidone (PVP), polyethylene glycol (PEG), and carboxymethyl cellulose (CMC) stabilized nZVI by reducing FeCl_3_ with hydrazine at the presence of PEG, CMC, or PVP [23]. Catalysts were active in the reduction of various aromatic ketones to produce aromatic alcohols as single products. The catalytic efficiency was in line with the size of nanoparticles, as smaller particles were more active, which was influenced by the capping material during the synthesis. The catalytic activity decreased in the order of Fe-PEG > Fe-CMC > Fe-PVP.

nZVI is known to catalyze carbon nanotube (CNT) growth. However, there is an upper limit for the size of the catalyst particles to nucleate single walled carbon nanotubes (SWCNTs), and the size of the nanoparticle determines the diameter of the tubes [25]. Li et al. synthesized uniform nZVI containing small amounts of molybdenum by thermal decomposition of metal carbonyls in octyl ether solution using a mixture of long-chain carboxylic acid and long-chain amine as protective agents. The sizes of NPs could be systematically varied from 3 to 14 nm by changing the experimental conditions. NPs were drop-dried on silicon wafers coated with thin alumina films and used as the catalyst for SWCNT growth by CVD with methane [25]. Small iron NPs can catalyze CNT growth during carbonization of organic materials in inert atmosphere. MWCNT growth was observed during the high-temperature carbonization of iron nitrate loaded styrene-based cation exchange resins due to the formation of small iron nanoparticles [51].

The Fenton’s reagent is widely used to degrade chemicals in water due to simplicity and low-cost. In the classic Fenton reaction, a combination of H_2_O_2_ and Fe^2+^ ions are used at low pH to produce hydroxyl radicals [58], which oxidize organic compounds; however, low pH has to be maintained to prevent Fe^3+^ precipitation and the concentration of H_2_O_2_ and Fe^2+^ are relatively high, which can inhibit organic material degradation due to radical scavenging [59]. The advantage of using nZVI in the Fenton reaction is that these limitations can be overcome, and nZVI is able to reduce oxygen on its surface leading to hydroxyl radicals, therefore, peroxide free Fenton-type reactions can be conducted for organic material degradation. There are several variants of this heterogeneous Fenton reaction, where the main role of nZVI is the generation of reactive radical species such as ∙OH, ∙O_2_^−^, ∙SO_3_^−^, and ∙SO_5_^−^. Leaching and precipitation of iron, however, cannot be avoided in these reactions what limits the reusability of the catalyst. nZVI is increasingly used for the development of the heterogeneous Fenton process for the treatment of complex wastewaters containing organic materials [60,61,62,63]. Note that details of catalytic heterogeneous Fenton reactions used for the degradation of organic molecules are provided below in Section 4 at the target pollutants. 

## 4. Removal of Toxic Pollutants from Water, Wastewater, Ground Water, or Soil/Sediments

**Figure 3:**
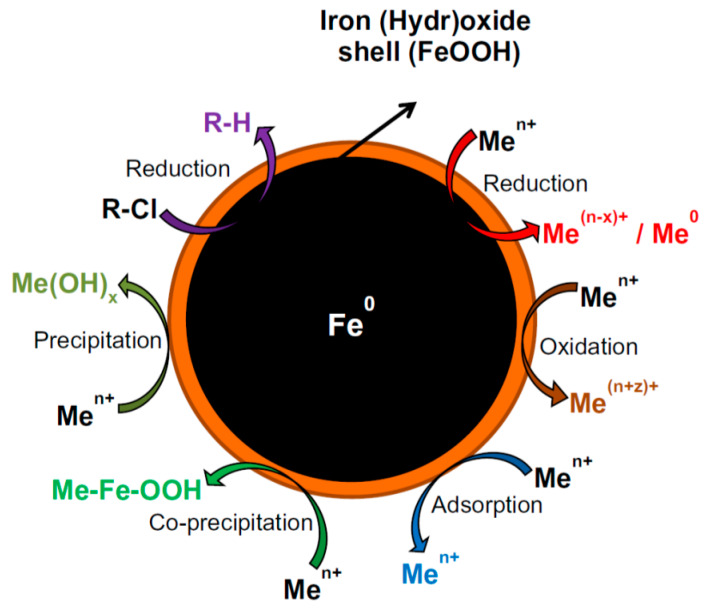
Various mechanisms for the removal of metals and chlorinated compounds from water. Reproduced with permission from [64]. Copyright Elsevier Inc., 2013.

**Figure 4:**
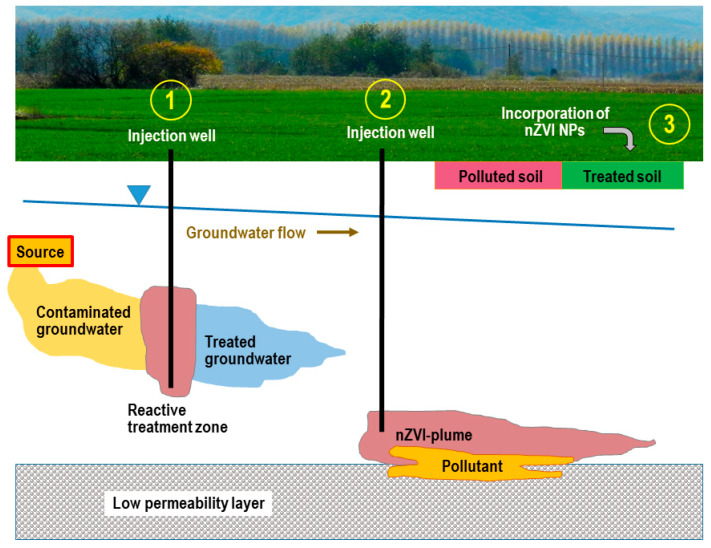
Technologies used to treat polluted groundwater and soils to adsorb or degrade pollutants: (1) injection of nZVI nanoparticles (NPs) to form a reactive barrier; (2) injection of mobile NPs to form an nZVI plume; and (3) incorporation of NPs into topsoil. Adapted from ref. [6].

Large amount and a wide variety of organic and inorganic compounds are produced in the chemical industry, many of them persistent, toxic, and non-biodegradable. Removing these compounds from industrial wastewaters, or especially from natural groundwater or soil if released is challenging. nZVI in an aqueous environment consists mainly of metallic iron core and a thin surface oxide layer and the core–shell structure provides combinational adsorption and reduction functions for contaminant removal and transformation. Organic and inorganic capping materials or supports of nZVI modify its properties. It is always a critical issue to find a balance between reduced reactivity and improved properties, such as mobility, adsorption capacity, and stability, due to capping/supporting. nZVI has a strong reducing power. It is mainly used in purification processes as a reductant, as a catalyst of heterogeneous Fenton oxidation at the presence of oxygen or hydrogen peroxide, adsorbent, or modifier of various adsorbents (Figure 3). The mobility of nZVI in water is less problematic if agitation can be applied, e.g., in laboratory or industrial wastewater treatment, but it is a serious issue in field applications, e.g., in soil remediation. nZVI has to be transported to contaminants at sufficient concentrations. Soil and groundwater contamination are closely linked; contamination in one affects the other. There are, in general, two possibilities for field applications either by creating a reactive zone with relatively immobile NPs or a reactive nanoparticle plume that migrates to contaminated zones. Topsoil applications are much more simple as conventional agricultural practices can be applied to distribute NPs to the surface of contaminated soil (Figure 4) [6].

### 4.1. Removal of Organic Compounds from Water and Soil

A large amount and a wide variety of organic compounds are used and produced in the industry for various purposes, such as stabilizers, preservatives, insecticides, pharmaceuticals, etc., just naming a few. Many of them are toxic and persistent in the environment if emitted. Our household is also a pollution source considering thousands of pharmaceuticals and personal care products, which are carelessly discarded into the nature. Contamination of surface water and ground water is emerging as a potential threat to the ecosystem and human health. There is a growing concern, for example, about antibiotics discharged into the environment, because these compounds or metabolites may remain active leading to bacterial resistance and deleterious effects on organisms [65,66]. nZVI is widely tested as a potential remediation agent for removing organic compounds from water. Removal efficiency of supported or unsupported nZVI toward organic materials is based on its adsorption and reducing power. nZVI is capable to reduce and modify many organic functional groups, thereby to transform toxic compounds to less harmful derivatives. The full degradation to carbon dioxide and water (mineralization), however, is not possible in an oxygen free environment. Organic pollutants can be mineralized using the nZVI catalyzed heterogeneous Fenton reaction. Note that chlorinated derivatives and dyes are discussed in Section 4.2. and Section 4.3.

#### 4.1.1. Removal of Organic Compounds from Water

Polycyclic aromatic hydrocarbons (PAHs) are typical environmentally persistent pollutants [67]. Li et al. synthesized nZVI NPs coated with silica and polydopamine (PDA) using a two-step process, and applied these composites as adsorbents for the removal of anthracene and phenanthrene from aqueous systems [68]. The adsorption process was found to be physical adsorption, and the nZVI/SiO_2_/PDA adsorbent exhibited good reusability as the adsorption efficiency barely decreased even after 10 cycles (Table 2).

Hu and Li prepared a thin aluminum hydroxide coating layer on nZVI NPs to increase the suspension stability and longevity of NPs in aqueous phase [26]. The coating increased the electrostatic repulsion and decreased the attraction between the nZVI/Al(OH)_3_ particles, therefore strongly increased suspension stability compared to bare nZVI. In addition, the Al(OH)_3_ coating shell effectively hindered aerobic corrosion of nZVI particles. nZVI/Al(OH)_3_ NPs were active in the reductive 4-nitrophenol removal from water.

He et al. prepared a nZVI/carbon core–shell nanocomposite containing small amounts of Pd by carbonizing a metal‒organic framework (MOF) built of iron and palladium ions and 2-amino-1,4-benzenedicarboxylic acid, and used it for catalyzing the degradation of phenol [50]. The carbon coat protected nZVI from corrosion and Pd accelerated the Fenton reaction. Small leaching of iron was detected, however, the amount was much smaller than in cases of nZVI NPs and commercial physically mixed Fe/C materials. A facile electrodeposition method has been developed by Xia et al. to synthesize dendritic micro-nano ZVI for the degradation of phenol [45]. Although this material showed good catalytic activity in the Fenton reaction, the iron leaching was higher than that of nZVI-MOF above, and the catalyst become covered during the reaction with Fe_3_O_4_ and degradation products; these latter reduced the activity of the nZVI catalyst in cycle experiments. Diao et al. applied the bentonite-supported nZVI/persulfate system for the simultaneous removal of phenol and of Cr(VI) from aqueous solutions [69]. A synergistic effect between Cr(VI) reduction and phenol oxidation was achieved, and Cr(VI) positively affected the oxidation rate of phenol, leading to catechol, 1,4-benzoquinone, propionic acid, and formic acid, as identified products. nZVI/bentonite was relatively stable after four cycles of reuse.

Bisphenol A (BPA) is used on a large scale as an intermediate in polymer production. Ma et al. developed a Fenton type reaction to decompose BPA in water, based on a nZVI/diatomite/organosilane composite that was prepared by grafting an acid precursor, 3-mercaptopropyl trimethoxysilane, onto diatomite [70]. The catalyst generates acid in situ by the reaction between the thiol group of the organic silane and H_2_O_2_, thereby maintaining the pH at an optimal value. The Fenton system exhibited 100% removal efficiency of BPA under natural pH conditions.

The steroidal estrogen 17α-ethinylestradiol (EE2) is a model endocrine disrupting chemical. Karim et al. investigated the degradation of EE2 by using commercial nZVI at pH 3, 5, and 7 under different oxygen conditions [59]. nZVI reduced EE2 directly in an oxygen free environment at all pH. The dominant radical, transforming EE2, was varied with pH at the presence of oxygen, namely ∙OH at pH = 3 and ∙O_2_^−^ at pH = 5. Interestingly, the degradation rate of EE2 was lower with larger amounts of oxygen, most likely due to faster nZVI oxidation and radical scavenging.

Machado et al. studied the degradation of amoxicillin (AMX), a common antibiotic, in water using green nZVI (sizes between 20 and 100 nm; irregular shapes) prepared from ammonium iron(II) sulfate with oak leaves extract [41]. nZVI was used as a reductant and in a separate experiment as the catalyst for the Fenton reaction. 100% degradation efficiency was achieved using an AMX/nZVI molar ratio of 1:15 when nZVI was applied as a reductant, and also when an AMX/H_2_O_2_/nZVI molar ratio of 1:13:1 was used in the Fenton reaction.

Chen et al. investigated the removal mechanism of the antibiotic metronidazole (MNZ) from deoxygenated water using PVP stabilized nZVI [71]. Although nZVI reacted with MNZ, no carbonization occurred and the total organic content hardly decreased in the solution. During the removal of MNZ, the nitro-group of MNZ was reduced into amino-group, and the byproduct 1-(2-hydroxyethyl)-2-methyl-5-aminoimidazole was present in the solution.

Ghauch et al. studied the removal of amoxicillin (AMX) and ampicillin (AMP) from water using bare, PEG, and zeolite stabilized nZVI [72]. nZVI/PEG was found to be more efficient than bare and zeolite supported nZVI. The antibiotics removal was attributed to a rapid rupture of the β-lactam ring (reduction), and a coprecipitation of decomposition products with iron corrosion products.

**Figure 5:**
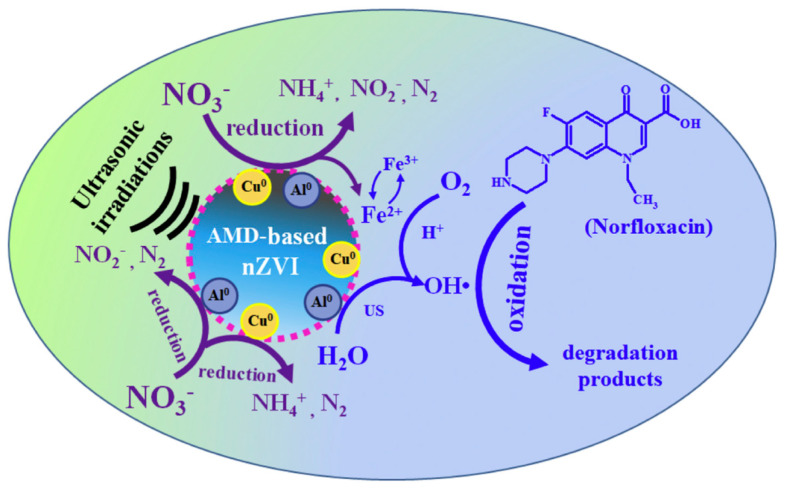
Possible mechanism for the simultaneous removal of nitrate and norfloxacin by acid mine drainage-based nZVI with ultrasonic irradiation. Reproduced with permission from [73]. Copyright Elsevier Inc., 2019.

Diao et al. synthesized nZVI from acid mine drainage, having high iron content and collected from a tailing pond of Dabaoshan sulfur-polymetallic mines, using sodium borohydride, and applied the prepared nZVI particles for norfloxacin antibiotic removal from water at the presence of nitrate and under ultrasonic irradiation [73]. nZVI particles contained also Cu and Al, although their presence could not be confirmed by X-ray diffraction, indicating amorphous state. nZVI effectively reduced both norfloxacin and nitrate, and ultrasonic irradiation enhanced the reaction rate through fluidizing and de-passivating nZVI. The norfloxacin decomposition and mineralization was proved to be based on the generation and Fenton type action of OH and O_2_^−^ radicals (Figure 5).

Tetracycline is a wide spectrum antibiotics and it is widely found in soil around the world due to municipal effluents and solid wastes. Chen et al. prepared polyvinylpyrrolidone (PVP) modified nZVI using the liquid-phase borohydride method for tetracycline removal from water [74]. Several degradation products were identified on both the composite surface and treated solution originating from tetracycline degradation via loss of N-methyl, amino, hydroxyl, carbonyl, and formyl groups, indicating that nZVI/PVP adsorbed both tetracycline and its degradation products. 

Metoprolol, a β-blocker, is an emerging persistent contaminant in aquatic environments. Daneshkhah et al. applied a heterogeneous Fenton system for metoprolol removal from water, based on sepiolite-supported nZVI prepared by the borohydride route [75]. A moderate removal rate of 67% was achieved.

#### 4.1.2. Removal of Organic Compounds from Spiked Soil

Soil remediation is a more challenging task than that of wastewater purification due to the complexity of the matrix. It has been observed so far that reagent requirements for soil remediation are much higher than those for the remediation of aqueous solutions [41].

Model laboratory experiments were conducted by Machado et al. for studying the removal of amoxicillin (AMX) antibiotic from a sandy contaminated soil using green nZVI as reductant and as the catalysts in the Fenton reaction [41] (Table 2). The sandy soil contained particles with diameters between 0.5 and 1 mm, did not contain detectable organic matter, and had a pH of 6.27. The soil was injected with aqueous AMX to cause contamination. Compared to the reference solution experiments the degradation efficiency for AMX was much lower, therefore the reagent requirements for soil remediation were more than 100-times higher than those for the aqueous solutions (see above); this is probably because of parallel reactions, limitations of the distribution, and agglomeration of NPs in the soil matrix.

Singhal et al. investigated the malathion ([(dimethoxyphosphinothioyl)-thio] butanedioic acid diethyl ester), a widely used insecticide, decomposition in soil under laboratory conditions [76]. The soil was contaminated for model experiments with a stock solution of malathion, then dried, crushed, and sieved. nZVI was mixed manually with the dry soil. The degradation of malathion with nZVI was effective, but degradation became slower as the NP size increased from 33 to 100 nm. Malathion was degraded to the non-toxic O-dimethyl phosphorodithioic compound.

### 4.2. Organic Dye Discolouration/Decomposition in Water

**Figure 6:**
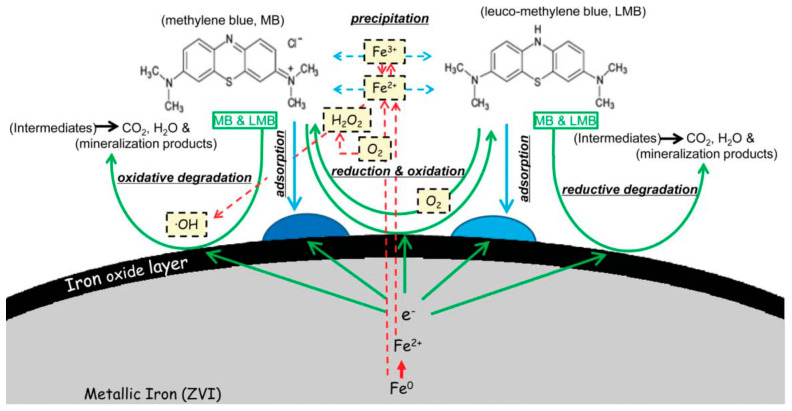
Possible mechanisms for the removal of methylene blue by ZVI from water. Reproduced with permission from [77]. Copyright Taylor and Francis Group, LLC, 2015.

Organic dyes are widely used in the chemical industry as colorants. Most of them are not biodegradable and toxic, therefore their removal from industrial wastewater is of crucial importance. Several model laboratory experiments were performed recently for studying the discoloration, degradation, and removal of organic dyes from water based on chemical reactions or physical adsorption processes (Table 3). The dye removal using nZVI is expected to proceed via the combination of adsorption, reduction, degradation, and/or precipitation processes depending on reaction conditions, such as pH of the solution and dissolved oxygen. Sun et al. observed, for example, that the methylene blue (MB) dye removal from water utilizes all four reaction path, depending on the pH of the solution and oxygen content [77] (Figure 6).

Kamali et al. have synthesized nZVI, with an average particle size of 27 nm, by the borohydride route and applied the NPs for the discoloration of methylene blue (MB) [78]. The pH of the solution was found to be a key factor for achieving 100% discoloration efficiency, as well as the high crystallinity and large specific surface area of iron NPs. Parlayici and Pehlivan have prepared a nZVI/Sycamore tree seed pod fibers (STSPF) composite by reducing iron ions adsorbed to porous STSPF using NaBH_4_, and applied nZVI/STSPF for the adsorption removal of MB, malachite green (MG), and methyl violet 2B (MV) from water [33] (Table 3). nZVI/STSPF was superior to STSPF in dye removal due to enhancements in STSPF pores by the modification with nZVI (size 23–41 nm).

The Fenton reaction is a powerful method to decompose organic molecules. Son et al. have synthesized an nZVI/montmorillonite nanohybrid and applied it in a heterogeneous Fenton reaction to decompose Rhodamine B (RhB) in water [79]. nZVI embedded in the montmorillonite matrix exhibited higher catalytic efficiency than reference commercial ZVI particles with an average diameter of 5 μm due to their small size and high surface area.

Yang et al. prepared nZVI with a thin Fe_2_O_3_ shell layer for a Fe@Fe_2_O_3_/NaHSO_3_ Fenton-like system to decompose Orange II dye [80]. The catalyst was found to be stable during the process even under strongly acidic conditions, and the Fe@Fe_2_O_3_/NaHSO_3_ system was more efficient than the traditional Fe@Fe_2_O_3_/H_2_O_2_. Rapid electron transfer was observed between the iron core and the oxide shell, which improved the oxidation efficiency.

Barreto-Rodrigues et al. optimized the reaction conditions of the nZVI synthesis from FeCl_2_ and NaBH_4_ (see above), and studied the degradation of the azo dye Disperse Red 1 (DR1) [18]. nZVI effectively decolorized DR1 in a short time, however, without mineralization, indicating that the degradation promoted by nZVI affected only the chromophore group (−N = N−). Mineralization was achieved upon Fenton oxidation by adding H_2_O_2_.

Xu et al. synthesized rGO/attapulgite-supported nZVI composites (rGO/APT-nZVI) to remove acid red 18 organic dye from aqueous solutions [81]. The composite effectively degraded the dye over a wide range of pH and kept degradation activity during a long storage. The removal process was based on two consecutive steps, namely the fast adsorption of the dye followed by the chemical degradation by nZVI. This latter resulted in smaller water soluble molecules due to splitting the dye at the N-N bond.

Bossa et al. used cellulose nanocrystals (CNC) to stabilize nZVI [82]. The mobility and reactivity of NPs were determined in sand/glassbead porous media and against methyl orange dye, respectively, and both were superior compared to properties of bare nZVI particles. CNC created a porous “mesh” around nZVI and increased the number of available reactive sites at the nZVI interface. It played a dual role as supporting matrix by limiting nZVI aggregation and improving mobility in porous media. It had low affinity for the glass bead surfaces. Wang et al. modified nZVI with hydroxyethyl cellulose and hydroxypropylmethyl cellulose as dispersants, and used the composites for organic dye (orange II, methyl orange, methyl blue, and methylene blue) discoloration [83]. Both the dispersity and antioxidizability of cellulose modified nZVI were better than those of bare nZVI. The discoloration followed a two-step mechanism, the adsorption of target dye and then the reduction of the −N = N− functional group of the dye.

Three, bentonite, kaolin, and native clay, supported nZVI composites were prepared by Kerkez et al. for the evaluation of oxidative degradation of the industrial azo dye Rosso Zetanyl B-NG [84]. All materials were effective in dye decolorization; this latter was significantly higher when heterogeneous Fenton system was used. The support material not only dispersed and stabilized nZVI but also improved the adsorption and degradation of the dye in Fenton oxidation. The decolorization efficiency was around 92% and the mineralization efficiency between 50% and 58% for all three composites.

**Table 3:** Organic dye decomposition using nZVI ^1^.

nZVI Cap/Support	Dye	pH, Reagent	Adsorption Capacity (mg/g)	Ref.
bare	Methylene blue	7.5	n.a.	[78]
STSPF ^2^	Methylene blue	5.0‒9.0	140.80	[33]
Cellulose	Methylene blue	5.96	n.a.	[83]
Cellulose	Methyl blue	5.96	n.a.	[83]
STSPF ^2^	Malachite green	5.0‒9.0	92.59	[33]
STSPF ^2^	Methyl violet 2B	5.0‒9.0	92.59	[33]
Montmorillonite	Rhodamine B	Air	n.a.	[79]
Fe_2_O_3_ shell	Orange II	3, air	n.a.	[80]
Cellulose	Orange II	5.96	n.a.	[83]
Bare	Disperse Red 1	3, H_2_O_2_	n.a.	[18]
rGO/attapulgite	Acid Red 18	2–8	400	[81]
Cellulose	Methyl orange	neutral	n.a.	[82]
Cellulose	Methyl orange	5.96	n.a.	[83]
Kaolin	Rosso Zetanyl B-NG	4.8	n.a.	[84]
Bentonite	Rosso Zetanyl B-NG	4.8	n.a.	[84]
Native clay	Rosso Zetanyl B-NG	4.8	n.a.	[84]

^1^ n.a. = not available; bare = NPs where a shell is not produced intentionally (contains a self-developed iron oxide layer); ^2^ sycamore (*Platanus occidentalis*) tree seed pod fibers (STSPF).

### 4.3. Degradation of Halogenated Organic Compounds 

Due to its reductive power, nZVI is capable of reducing halogenated organic compounds by reductive dehalogenation, leading to hydrocarbons and halide ions (Equations (4) and (5)). The process may be divided into two steps, namely (i) adsorption onto the iron surface and (ii) reduction via hydrogenolysis or dehalogenation [64]. In hydrogenolysis, a chlorine atom is replaced by a hydrogen atom and requires an electron donor, Fe, as well as a proton donor. In reductive elimination halogen atoms are released without the addition of hydrogen and requires the iron and the presence of at least two halogen atoms in α or β position. α-elimination leads to the formation of a carbene and thus to a consecutive fast dimerization. β-elimination leads to unsaturated organic compounds. An illustrative example is the γ-hexachlorocyclohexane dechlorination to benzene [85].

Fe^0^ + H^+^ + R-X → R-H + Fe^2+^ + X^−^ (X = F, Cl, Br, I) (4)

Fe^0^ + H^+^ + 2 R^1^-X → R^2^ + Fe^2+^ + 2X^−^ (5)

Although it is the major aim in detoxification processes to decompose toxic halogenated compounds, and nZVI is capable to do this, it is also a significant concern if daughter products are more toxic than the parent halogenated compounds. A full decomposition of halogenated organic compounds (demineralization) to carbon dioxide, water, and halide is not possible using only nZVI, but at the presence of oxygen or hydrogen peroxide, iron catalysis the Fenton oxidation leading to demineralization. This latter, however, is not possible in field applications.

#### 4.3.1. Removal of Halogenated Compounds from Water and Wastewater

Perfluorinated compounds are extremely persistent pollutants, detected worldwide, due to their stability to metabolic and environmental degradation [86,87]. Arvaniti et al. studied the removal of perfluorooctanoic acid, perfluorononanoic acid, perfluorodecanoic acid, and perfluorooctane sulfonate from water using Mg-aminoclay coated nZVI [86] (Table 4). This composite was found to be much more effective than commercial air stabilized nZVI or freshly synthesized uncoated nZVI. The removal ability, however, slightly decreased when three days old nZVI was used. The pollutant removal of nZVI/Mg-aminoclay was based on both partial sorption and degradation. No organic byproducts were detected, indicating full carbonization, which was in line with the identification of fluoride ions. Liu et al. applied cetrimonium bromide stabilized nZVI to remove perfluorooctanic acid from groundwater at acidic pH with or without the addition of H_2_O_2_ [69]. NPs showed effective removal for perfluorooctanic acid under visible light, but higher removal was obtained under extreme acidic environment. In this latter, the addition of H_2_O_2_ increased the removal efficiency.

Halonitromethanes, a class of water disinfection byproducts in drinking water, are of emerging health concerns. Chen et al. synthesized nZVI/graphene composite by carbonizing a glucose–ferric chloride mixture in a hydrogen–argon atmosphere, and applied the composite for trichloronitromethane removal from water [88]. The composite effectively dechlorinated and denitrated trichloronitromethane to methylamine. Dichloronitromethane as a dechlorination intermediate was detected. The reusability of the composite gradually decreased in consecutive cycles due to oxidation of iron and surface passivation, however, Fe^0^ could be restored again by calcination.

Li et al. prepared nZVI with sizes between 10 and 50 nm by precision ball milling, and tested this material through reactions with a mixture of seven model chlorinated aliphatic compounds, namely 1,1-dichloroethane, 1,1,1-trichloroethane, tetrachloromethane, 1,2-dichloroethane, trichloroethene, tetrachloroethene, and 1,1,2,2-tetrachloroethane [12]. Ball milled nZVI was found to be more reactive than nZVI prepared by the borohydride solution method due to larger surface area and fresher surface, and effectively dechlorinated all seven chlorinated hydrocarbons. Ribas et al. produced highly reactive nZVI via wet milling through abrasion by alumina, and applied these NPs in trichloroethylene and tetrachloroethylene removal from water [13]. The removal capacity was observed to be higher than that of commercial nZVI particles, possibly related to the absence of a thick and continuous oxide layer on the surface and high density of defects in the highly-deformed nanostructure of the milled samples. Gao et al. produced nZVI/carbon composite by ball milling microsize iron and activated carbon powders [15]. Iron NPs were dispersed in activated carbon. This composite material instantaneously sorbed trichloroethene from aqueous solutions and subsequently decomposed it into non-chlorinated products, preferably to ethene. The nanocomposite dechlorinated trichloroethene at a slightly slower rate than milled iron NPs.

nZVI could be prepared by reducing iron salts with sodium dithionite at high pH, resulting in a mixture of very small elemental iron particles imbedded in a sulfite hydrate crystal matrix [24]. These nZVI NPs were shown to be slightly more effective in trichloroethylene dechlorination than those prepared by the borohydride method.

Lindane (γ-hexachlorocyclohexane) is a persistent, formerly widely used pesticide. Elliott et al. investigated the dechlorination of lindane in water and water–ethanol mixture [85]. nZVI was found to be more effective in dechlorination than microscale iron, and reduced lindane to benzene. A key reaction intermediate, γ-3,4,5,6-tetrachlorocyclohexene, was also identified among dechlorination products.

Chloramphenicol is a broad-spectrum antibiotic with excellent antibacterial properties, however, it has various side effects. Therefore, its appearance in river waters and municipal sewage treatment plant effluents is of emerging concerns. Xia et al. investigated the chloramphenicol removal from water using nZVI [89]. nZVI effectively reduced chloramphenicol, and the reduction was shown to proceed via dechlorination followed by nitro group reduction. Florfenicol is a widely used antibiotics in human medicine and aquaculture. Cao et al. applied sulfide stabilized nZVI for florfenicol removal from spiked natural waters, namely groundwater, river water, seawater, and wastewater [90]. Sulfidized nZVI was prepared by treating an iron sulfate solution with an aqueous mixture of NaBH_4_ and Na_2_S_2_O_4_. It was observed that the reactivity of sulfidized nZVI was relatively unaffected either by dissolved ions or organic matter in the waters tested. The composite was effective in florfenicol reduction; consecutive dechlorination, dechlorination, and defluorination of florfenicol led to four degradation products, namely C_12_H_15_ClFNO_4_S, C_12_H_16_FNO_4_S, C_12_H_17_NO_4_S, and C_12_H_17_NO_5_S.

Diazepam (DZP) is a commonly used drug and found in sewage treatment plant effluents and in river and potable water. Bautitz et al. applied a heterogeneous Fenton system for the degradation of diazepam [66]. The nZVI/EDTA/O_2_ system effectively degraded and mineralized diazepam when nZVI was pretreated with sulfuric acid to remove the oxide layer before the reaction. No mineralization was observed under nitrogen atmosphere.

Organophosphorus insecticides, due to the direct carbon to phosphorous covalent bond, are resistant to chemical and thermal degradation. A simple and rapid method was developed by Mehrotra et al. for the catalytic degradation of dichlorvos (2,2-dichlorovinyl dimethyl phosphate) insecticide based on biosynthesized protein-capped nZVI catalyzed Fenton reaction [44]. Yeast extract was used as a reducing and capping agent, and the synthesized small NPs, in the range of 2–10 nm, effectively catalyzed the carbonization of dichlorvos to phosphate, chloride, carbon dioxide, and water.

Polybrominated diphenyl ethers, extensively used as brominated flame retardants, were proved to be toxic to the human nervous, endocrine, and immune systems [91]. Tan et al. developed an effective method to degrade and remove decabromodiphenyl ether from water [91]. The debromination and ring-opening of decabromodiphenyl ether was realized by a Fenton-like degradation process using nZVI supported on Fe_3_O_4_ NPs. A 100% removal efficiency and 80% degradation efficiency of decabromodiphenyl ether was achieved by the composite accompanied with ultrasound. Tan et al. investigated the solvent effect and the kinetics of the degradation of decabromodiphenyl ether in mixed water/THF solvent [92]. THF inhibited the degradation, which is related to the observation that water played a role as hydrogen donor during the reductive degradation. Bromamine acid is a highly water-soluble and non-biodegradable chemical intermediate, widely used in the chemical industry. The degradation of bromamine acid in water was investigated by Fei et al. using sepiolite supported nZVI [93]. nZVI/sepiolite NPs were more stable in air and possessed better water solubility than bare nZVI, and showed high activity in the degradation of bromamine acid.

Capping layer on nZVI strongly influences the reactivity of NPs. Schmid et al. investigated the reactivity of three types of commercially available nZVI (bare and polyacrylic acid (PAA)-coated with magnetite shell, and one having FeO-Fe_3_O_4_ double shell) with iopromide (1-*N*,3-*N*-bis(2,3-dihydroxypropyl)-2,4,6-triiodo-5-(2-methoxyacetamido)-1-*N*-methylbenzene-1,3-dicarboxamide, a contrast agent in radiography [94]. All these composites quickly dehalogenated iopromide, producing iodide. However, nanoparticles with thin Fe_3_O_4_ coating were the most reactive, and the easy-to-handle and air-stable particles with double FeO-Fe_3_O_4_ coating were the least reactive.

**Table 4:** Removal of halogenated organic compounds from water and soil ^1^.

nZVI Cap/Support	Pollutant	pH, Reagent	Degradation Product	Ref.
Mg-aminoclay	Perfluorooctanoic acid	3	Not detected	[86]
Mg-aminoclay	Perfluorononanoic acid	3	Not detected	[86]
Mg-aminoclay	Perfluorodecanoic acid	3	Not detected	[86]
Mg-aminoclay	Perfluorooctane sulfonate	3	Not detected	[86]
CTAB	Perfluorooctanoic acid	0.5	n.a.	[87]
Graphene	Trichloronitromethane	6.5	Methylamine	[88]
Fe_2_O_3_	Chlorinated hydrocarbons ^2^	Neutral	n.a.	[12]
MEG	Chlorinated hydrocarbons ^3^	Neutral	n.a.	[13]
Carbon	Trichloroethene	7	Ethene	[15]
Sulfite hydrate	Trichloroethylene	Neutral	n.a.	[24]
FeO	Lindane	5–9	Benzene	[85]
Bare	Chloramphenicol	6.8	C_11_H_16_N_2_O_3_	[89]
Sulfide	Florfenicol	7	C_12_H_17_NO_4_S ^4^	[90]
Bare	Diazepam	2.2	n.a.	[66]
FeOOH/protein	Dichlorvos	Neutral, H_2_O_2_	PO_4_^3−^, Cl^−^	[44]
Fe_3_O_4_	Decabromodiphenyl ether	7.1, H_2_O_2_	CO_2_, H_2_O ^5^	[91]
Sepiolite	Bromamine acid	3–11	n.a.	[93]
Fe_3_O_4_	Iopromide	7.2–7.9	n.a.	[94]
PAA/Fe_3_O_4_	Iopromide	7.2–7.9	n.a.	[94]
FeO/Fe_3_O_4_	Iopromide	7.2–7.9	n.a.	[94]

^1^ n.a. = not available; bare = NPs where a shell is not produced intentionally (contains a self-developed iron oxide layer); CTAB = cetrimonium bromide; MEG = monoethylene glycol; ^2^ Mixture of 1,1-dichloroethane, 1,1,1-trichloroethane, tetrachloromethane, 1,2-dichloroethane, trichloroethene, tetrachloroethene, and 1,1,2,2-tetrachloroethane; ^3^ Mixture of trichloroethylene and tetrachloroethylene. ^4^ Degradation products: C_12_H_15_ClFNO_4_S, C_12_H_16_FNO_4_S, C_12_H_17_NO_4_S, and C_12_H_17_NO_5_S; ^5^ Large number of intermediates were identified.

#### 4.3.2. Removal of Halogenated Compounds from Groundwater and Soil 

One of the major challenges of groundwater and soil remediation in field applications is the transportation of injected nZVI to required distances at sufficient concentrations, or to create a reactive barrier zone right in contaminated groundwater stream.

Wei et al. demonstrated the effectiveness of nZVI in groundwater remediation using a 200 m^2^ pilot-scale field test located in the downstream ground water direction of a vinyl chloride monomer plant [95]. The groundwater was highly contaminated with chlorinated organic compounds, especially with vinyl chloride. Both commercial and borohydride method synthesized nZVI were used and were found to be effective in vinyl chloride dechlorination. nZVI was observed to be mobile in the aquifer, exhibiting an effective travel distance of at least 3 m. An increase in total iron, total solid, and suspended solid concentrations were also observed in the groundwater due to nZVI migration. The vinyl chloride degradation efficiency was greater than 90% in both upper and middle soil layers, but was much smaller in the bottom layer, possibly due to the smaller nZVI concentration in the latter (Table 5).

Benneth et al. investigated the mobility and reactivity of carboxymethyl cellulose (CMC) stabilized nZVI, prepared by the borohydride route, in saturated sediments of an existing aerospace facility contaminated with tetrachloroethene (PCE) and trichloroethene (TCE) [96]. CMC/nZVI NPs were injected into depth-discrete aquifer zones, and were observed to be mobile in the aquifer but gradually lost mobility in 13 h, possibly due to interactions with sediments. Rapid abiotic degradation of chlorinated ethenes to ethene occurred upon nZVI injection, however, the total dechlorinated ethane mass was low, possibly due to the lack of substantial mixing between injected solutions and contaminated groundwater. Quinn et al. applied vegetable oil emulsified nZVI for the treatment of the soil of an abandoned air force station contaminated with TCE [97]. Although significant quantities of TCE degraded, the effectiveness of the remediation suppressed, because NPs were not evenly distributed within the target treatment area, not migrated as far as expected from the injection points, and migrated up above the target treatment depth. TCE concentration was reduced significantly in the soil and groundwater, but increasing concentrations of intermediate dechlorination products, namely *cis*-dichloroethene and vinyl chloride, was detected in the groundwater. As the follow up of these field tests O’Hara et al. conducted a series of laboratory experiments to provide insight into the mechanisms responsible for the decrease of TCE concentrations in field applications [98]. Laboratory tests suggested that the decrease in TCE in the emulsified nZVI treatment is due to both the abiotic degradation of the TCE associated with nZVI and due to the sequestration of the TCE into the oil. Consequently, emulsified nZVI was able to reduce TCE concentrations to lower levels than that obtained by nZVI alone.

Several laboratory studies have been performed to date on spiked soil samples to test the ability of nZVI to remove halogenated organic compounds. Khuntia et al. synthesized well-defined monodisperse pectin-capped nZVIs with an average diameter of 25 nm by the borohydride route, and employed NPs to degrade DDT (dichlorodiphenyltrichloroethane) in spiked soil [30]. The soil was incubated with nZVI suspension and the decomposition was studied for 28 days. nZVIs were capable of degrading DDT, and the degradation efficiency was much larger than that of reference macrosized ZVI.

Remediation of organic contaminant source zones is technically challenging. The applicability of nZVI flakes in perchloroethylene contaminated soil remediation was demonstrated by Köber et al. at a site formerly used as a dry cleaning facility and is highly contaminated with perchloroethylene (PCE) between 10 and 14 m below ground [14]. nZVI flakes were manufactured by milling, and inserted into quaternary sand layers to degrade perchloroethylene. The radius of influence was about 1 m. Iron flakes remained reactive for more than 1 year; the perchloroethylene concentration strongly decreased and ethene was produced in high concentrations. Jordan et al. applied nZVI in a suspension with food grade inorganic material to treat PCE contaminated soil beneath and adjacent to a former dry cleaner [99]. nZVI injections resulted in an about 2.5 m NP distribution around the injection points and reducing conditions in the treatment zone, but no noticeable effect on groundwater flow patterns. Complete removal of PCE was achieved in three months, but high concentration of *cis*-dichloroethylene and vinyl chloride persisted, although with a declining trend. Ethene formation sustained for over 1.5 years.

### 4.4. Heavy Metal and Metalloid Ion Removal 

Heavy metals and metalloids are widely used in the industry, e.g., in smelting, electroplating, and manufacturing, and wastewaters of such industrial plants often contain a complex and varied mixture of heavy metal ions. Heavy metal pollution is one of the most serious concerns of industrial activity, therefore effective cleaning technologies are required. nZVI particles have a core–shell structure, which enables them to behave as an electron source (core) and a site for surface complexation (shell). Precipitation on the surface is the most common sequestration mechanism of metal ion removal by nZVI. The interaction between nZVI and metal and metalloid ions can be categorized [64,100] as:

1. Reduction (examples: Cr, As, Cu, U, Pb, Ni, Se, Co, Pd, Pt, Hg, and Ag ions),
2. Adsorption (examples: Cr, As, U, Pb, Ni, Se, Co, Cd, Zn, and Ba ions),
3. Oxidation/reoxidation (examples: As, U, Se, and Pb ions),
4. Coprecipitation (examples: Cr, As, Ni, and Se ions),
5. Precipitation (examples: Cu, Pb, Cd, Co, and Zn ions).

In many cases a combination of some of these processes occurs depending on the redox potential of the system (oxidized/reduced form of the metal or metalloid) and capping layer of the nZVI. Systems having far more positive potential than Fe^2+/3+^/Fe are typically reduced and the reduced form can precipitate on or close to the nZVI surface (typical examples are Cr, Hg, Ag, As, Cu, U, and Se ions). Metal ions of systems with just slightly more positive redox potential than that of Fe^2+/3+^/Fe can be both reduced and adsorbed (examples are Pb and Ni ions). Metal ions of systems with similar or more negative redox potential than that of Fe^2+/3+^/Fe are mostly adsorbed on the oxide/hydroxide or capping layer of nZVI without reduction (examples are Cd and Zn ions). Due to the redox reaction iron is leaching into the environment, unless it is coprecipitated in the form of hydroxide depending on the pH of the medium. Han et al. pointed out, for example, that the total iron dissolved into the aqueous phase during Cu(II) removal correlated linearly with the amount of Cu(II) being removed [19].

#### 4.4.1. Removal of Heavy Metals and Metalloids from Water and Wastewater

Arsenic contamination in groundwater, due to its high toxicity, is one of the major concerns in many countries for producing clean drinking water for the population [101]. It exists in water mainly in the form of arsenite, As(III), and arsenate, As(V). To remove arsenic from water, Liu et al. synthesized an adsorbent material by anchoring nZVI onto the surface of graphene-silica composites (GS) using the borohydride route [101]. The composite exhibited great potential to remove both As(III) and As(V) and regeneration ability, however, the adsorption performance highly depended on the pH of solutions (Table 6). Both adsorption and electrostatic interactions play a role in As removal from water. Wu et al. demonstrated that the corrosion of nZVI plays a more important role in As(V) adsorption on nZVI surface than electrostatic interactions [102]. With increasing solution pH, γ-FeOOH is decreasing and Fe_3_O_4_/γ-Fe_2_O_3_ is increasing on the nZVI surface, and the latter has stronger adsorption ability to As(V). This compensates the negative effect of electrostatic repulsion at higher pH, therefore nZVI could be used for removing As(V) at a broad pH range. Na-Montmorillonite-supported nZVI was synthesized by Bhowmick et al. to remove As(III) and As(V) from aqueous solutions under oxidative atmosphere [103]. Reduction of adsorbed As(V) was not observed on the surface, however, complete oxidation of As(III) to As(V) took place possibly due to Fenton-type reaction via oxidizing intermediates formed from air oxygen. Competing anions like sulfate, nitrate, and bicarbonate did not show a pronounced effect on adsorption, but phosphate had an inhibitory effect due to competition for adsorption sites. Tanboonchuy et al. studied the arsenic removal from water using bare nZVI [104]. Experiments indicated that the removal of As(V) was easier than that of As(III), and the presence of oxygen helped to remove arsenic through oxygen-induced corrosion products of iron. Interestingly, the total arsenic concentration was enhanced when both As(III) and As(V) were present compared to cases when As(III) or As(V) was alone. 

Yan et al. demonstrated the multi-tiered distributions of arsenic species in the nanoparticles after reactions between nZVI and arsenite; As(V) was concentrated at the exterior particle surface, whereas As(0) was enriched at the metallic iron/oxide interface, suggesting that As(III) oxidation and reduction may occur in parallel at different subdomains of the nanoparticles owing to the particle’s core–shell structure [105]. Real systems usually contain large number of ions, and some of them may compete with the target contaminant ions for adsorption sites of the adsorbing material. Suazo-Hernández et al. investigated the removal of As(V) from water in the presence of selenate (Se(VI)) using nZVI and nZVI supported on zeolite [106]. The As(V) removal efficiency of these adsorbents was high with a minimal competition effect of Se(VI). The As(V) removal capacity was higher for nZVI/zeolite than that of nZVI. 

**Figure 7:**
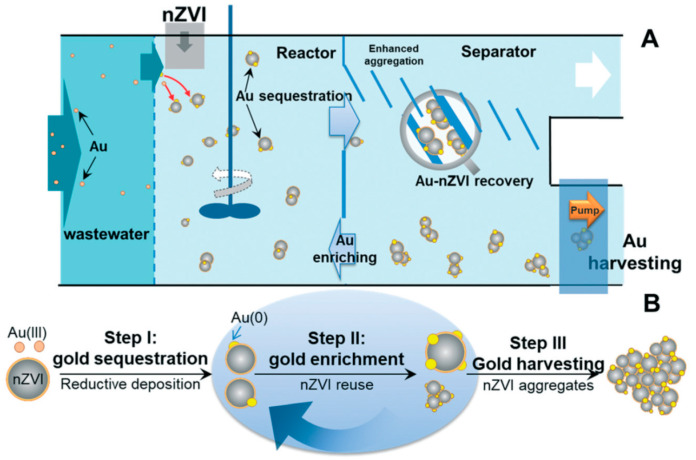
(**A**): A model of gold recovery from wastewater using nZVI and (**B**): the schematic process of gold recovery using nZVI. Reproduced with permission from [107]. Copyright The Royal Society of Chemistry, 2019.

Due to leakage or accidental release, wastewater from electroplating and hydrometallurgical gold smelting often contains gold ions whose recovery is important from both an environmental and economical point of view. Li et al. prepared nZVI, containing 78% Fe^0^ and a capping oxide layer, by the borohydride route and tested the capability of nZVI to remove gold ions from water under laboratory conditions [107]. nZVI was very effective; the gold ion concentration in treated water could be reduced below 0.1 μg L^−1^ (ppb). For larger scale applications, nZVI was synthesized via high-energy precision milling and applied for gold removal from wastewater of a smelting factory containing 37 ppb gold on average [107]. The schematics of the recovery plant and the process are illustrated in Figure 7. The continuously operating plant could recover over 5000 g gold in a year with reducing the gold content in wastewater below 0.2 ppb.

Cadmium is considered to be one of the highly toxic heavy metals, which is difficult to be metabolized after entering the environment. Zhu et al. synthesized a potassium carbonate activated porous straw biochar (KBC)/nZVI composite applying the borohydride reduction method and studied the Cd(II) removal efficiency of nZVI/KBC from water [108]. The synergistic effect between nZVI and KBC was found to be very strong, and the adsorption capacity increased compared to KBC. The adsorption process was the result of surface complexation of Cd(II) ions, partial reduction of these latter by nZVI, and surface precipitation of iron ions. The specific surface area of the adsorber was not the decisive factor for the adsorption of Cd(II). The advantage of using nZVI modified biochar instead of biochar in Cd(II) removal was also emphasized by Saffari; the composite of pistachio residues biochar and nZVI exhibited higher Cd(II) removal efficiency from aqueous solutions than the unmodified biochar [109]. In order to increase stability and reactivity of iron nanoparticles, nZVI supported on reduced graphene oxide (rGO) was prepared by Li et al. from Fe(III) adsorbed on GO using a H_2_/Ar plasma reduction method [49]. The composite exhibited high adsorption capacity for Cd(II) removal from water, higher than that of reference nZVI or rGO, and could be regenerated by the plasma reduction technique. It did not show any obvious decrease in efficiency after four adsorption–desorption cycles.

Cobalt is among the elements encountered in industrial wastes. Although, cobalt is an essential element for life in very small amounts, higher concentrations or ingestion is a health risk. Uzum et al. developed a kaolinite-supported nZVI adsorbent to remove Co(II) ions from water [110]. nZVI/kaolinite demonstrated high Co(II) removal abilities, and the removal mechanism was confirmed to be chemical complexation, to the exposed hydroxyl groups of NPs, and precipitation at high metal ion concentrations. Xing et al. deposited nZVI onto rGO surface by reducing a mixture of GO and ferrous ions with sodium borohydride and applied the composite for Co(II) removal from water [111]. The adsorption capacity of the adsorbent was low at the acidic pH, but sustained similar high value in a wide pH range of 4.0‒9.0. The adsorption of Co(II) on nZVI/rGO composite was attributed to inner-sphere complexation and dissolution/reprecipitation of the substituted metal oxides.

Chromium is extensively used in various industrial processes, e.g., in metallurgy, leather tanning, electroplating, etc. It mainly exists in the form of Cr(VI) and Cr(III) in the environment. Cr(VI) is toxic, carcinogenic, and has a high mobility in aqueous environment. Cr(III) on the other hand is a nontoxic and necessary trace element for mammalian metabolism, and it is easy to precipitate and remove Cr(III) from solutions. Removing and/or reducing Cr(VI) from natural and industrial wastewaters is of fundamental importance. Shu et al. used nZVI modified biochar, prepared by carbonizing almond shell and depositing nZVI by the borohydride method, for Cr(VI) removal from water [112]. The pH had a key role and acidic conditions favored the chromium removal. The biochar itself had a much lower adsorption capacity than nZVI/biochar composite, which indicated that adsorption cooperated with the redox reaction to remove Cr(VI) from water. In the removal process, Cr(VI) was adsorbed by nZVI/biochar and reduced to Cr(III) by nZVI. Chi et al. applied lignin as the stabilizer and Al-bentonite as the carrier for nZVI to enhance its Cr(VI) detoxification performance in water [113]. This composite was more efficient in Cr(VI) removal than bare nZVI and Al-bentonite supported nZVI, and the synergistic effect of stabilizer and carrier could alleviate the deposition of redox byproducts on the surface of iron NPs, thereby reducing its deactivation and improving its reusability. Vilardi et al. applied CMC stabilized and MWCNT supported nZVI prepared by the borohydride solution technique for Cr(VI) removal from water [114]. nZVI/CMC showed better Cr(VI) removal efficiency than nZVI/MWCNT, and both of these composites performed better than pure MWCNTs. The predominant Cr(VI) removal mechanism was the reduction of Cr(VI). In order to enhance the dispersibility and stability of nZVI in the air, Liu et al. applied porous pumice rock as the support material for nZVI [27]. Pumice was effective to prevent nZVI particles from agglomerating, and NPs were uniformly distributed on the surface. Mucha et al. immobilized nZVI on electrospun carbon nanofibers (ECNFs) surface using the borohydride reduction method and applied nZVI/ECNF in Cr(VI) removal from water. nZVI/ECNF exhibited far better capacity and faster rate to remediate Cr(VI) contaminated water than bare nZVI [115].

**Figure 8:**
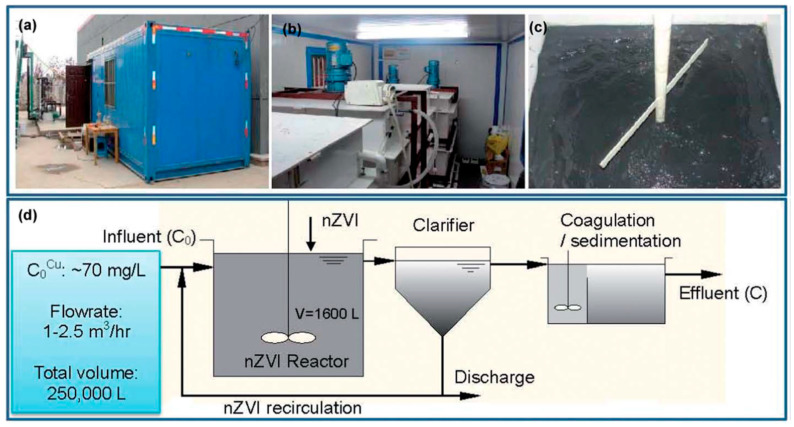
(**a**) Shipping container of the pilot reactors; (**b**) the nZVI reactor; (**c**) the nZVI suspension in the reactor (C_0_ is the concentration of copper in the wastewater); and (**d**) a process flow chart of the pilot test. Reproduced with permission from [119]. Copyright The Royal Society of Chemistry, 2014.

Copper is an essential trace element for living organisms, but high copper levels can cause detrimental health effects. It is one of the most common pollutants in industrial effluents, e.g., of power stations, electroplating, combustion, mining, and smelting. Both unsupported or supported nZVI can effectively remove Cu(II) from wastewaters by a redox mechanism, leading to the formation of Cu_2_O and Cu^0^ [110,116,117]. Xiao et al. fabricated MWCNT enforced polyacrylic acid (PAA)/polyvinyl alcohol (PVA) nanofibrous mats by electrospinning and used it as the support for nZVI particles deposited by the borohydride route [118]. The results indicated that Cu(II) removal occurred via chemical reduction and deposition on the nZVI NP surfaces to form the Fe/Cu alloy. Neither copper nor iron ions were released into the solution after the sorption of copper on the Fe-containing mats. Li et al. demonstrated the effectiveness and feasibility of nZVI application on the industrial scale by treating wastewaters of a printed-circuit board manufacturing plant containing high levels of Cu(II) [119] (Figure 8). In a continuous operation, 250,000 L of wastewater containing 70 mg L^−1^ Cu(II) was treated with 55 kg nZVI prepared by the borohydride route. Greater than 96% removal efficiency was achieved, and the end product was a valuable composite of iron and copper (20%–25%), which can partially offset the treatment costs.

Mercury ions are highly toxic materials therefore they have to be removed from industrial wastewater before water is discharged to the environment. Liu et al. synthesized nZVI supported on pumice using the liquid-phase borohydride method for the removal of Hg (II) from aqueous solution [27]. Hg(II) were removed by a rapid physical adsorption at the beginning of the process and predominantly by reduction. The adsorbent could be regenerated and a high removal rate could be maintained on the second, third and fourth runs. 

Nickel compounds are classified as human carcinogens. Li and Zhang prepared nZVI with spherical particle size of around 50–70 nm in solution using NaBH_4_ and applied it for Ni(II) ion removal from water [120]. NPs had a core–shell structure with thin FeOOH layer formed during the synthesis. During the removal process, Ni(II) first quickly formed a surface complex with nZVI, and then it was reduced to metallic nickel in the consecutive step.

Lead is widely used in the industry, e.g., in battery production, casting, electroplating, and metallurgy, and some of these industrial processes discharge lead-bearing wastewater, that has to be purified due to the high toxicity and non-biodegradability of lead. Zhang et al. synthesized spherical core–shell nZVI with an average size of 55 nm through NaBH_4_ reduction and studied the lead ion removal efficiency of dried and undried NPs from water [22]. The removal efficiency of undried nZVI was found to be about 14.2% higher than that of dried nZVI pointing out the importance of nanoparticle treatment during the synthesis. Undried nZVI had a relatively lower degree of oxidation on the surface than dried nZVI, and hence had more active sites. A nZVI/Mg(OH)_2_ composite with exceptionally high lead adsorption capacity, resulted from a synergistic effect of Mg(OH)_2_ and nZVI, was prepared by Liu et al. [121]. The lead removal process is based on Pb(II) adsorption by Mg(OH)_2_, Pb(II) reduction by nZVI, and Pb(II) precipitation as Pb(OH)_2_. The composite was successfully applied to remove Pb(II) from acid Pb−Zn mine tailing leachate. Kim et al. used naturally occurring zeolite to support nZVI for Pb(II) removal [122], and the removal was observed to largely depend on the solution pH and temperature.

Selenium is an essential micronutrient for humans and animals, but in high concentrations it is toxic and teratogenic, and considered to be a dangerous pollutant for the environment. Selenium is mostly found as selenite, Se(IV), and selenate, Se(VI), in aqueous media. Reduced forms of selenium, Se(0) and Se(II), are insoluble in water and thus less bioavailable, therefore reduction of soluble highly oxidized forms of selenium is a possible strategy for Se(IV/VI) removal from water [123]. Vilardi et al. investigated the Se removal efficiency of nZVI/CMC and nZVI/MWCNT using an atomic absorption stock solution as the model Se contaminated wastewater [114]. nZVI/MWCNT performed better than nZVI/CMC or bare MWCNTs. NPs size strongly influenced the adsorption efficiency as smaller NPs were more effective; sonication during the synthesis was found to be essential to obtain unimodal size distribution and small particles. Adsorption was found to be the main removal process for Se using these nZVI composites. Ling et al. investigated the selenium removal from water and the mechanisms of intraparticle reduction of Se(IV) [9]. It was observed that chemical reduction of Se(IV) to Se(0), and encapsulation into the NPs are the main chemical processes. These reduction and sequestration mechanisms explained the rapid Se(IV) removal and large capacity of nZVI for selenium encapsulation. Gui et al. developed nZVI-iron oxide core–shell NPs-functionalized polyvinylidene fluoride (PVDF)/polyacrylicacid (PAA) membranes to remove selenite and selenate from coal-fired power plant scrubber water [124]. Sulfate and chloride decreased the reaction rate of selenium removal by lowering the reactivity of iron due to competitive adsorption and iron corrosion, but this was overcome by combining nanofiltration and the iron-functionalized membranes. In addition to selenium removal, the developed iron-functionalized membrane reduced the concentration of other toxic metal and metalloid ions in the scrubber water such as arsenic, nickel, and mercury, as well as nitrate.

Uranium is a common contaminant in soil and groundwater near uranium mining and processing, and can be found in drinking water wells due to naturally occurring sources. Uranium exists mainly as U(VI) and U(IV) species in the natural environment; U(VI) is highly mobile and toxic and dominates under oxidizing conditions, U(IV) is less soluble than U(VI) species with low toxicity under reducing conditions [125]. Due to its high biological toxicity, uranium removal from the environment is important. Reducing soluble U(VI) to the less soluble U(IV), in principle, is a possible remediation method for contaminated sites. Li et al. found that nZVI and nZVI/rGO obtained according to a NaBH_4_ reduction method are very effective reactive materials in uranium removal under anoxic conditions [126]. The removal rate by nZVI/rGO was observed to be faster than that by nZVI, but the adsorption capacity of nZVI was larger. The former could be explained by the larger surface area, oxygen-containing functional groups of rGO, and improved suspension stability of the composite, and the latter by the smaller iron content in mass unit. Uranium immobilization was due to the rapid reductive precipitation of U(VI) to the sparingly soluble U(IV) species. Uranium removal was independent of pH, the presence of carbonate ions, humic acid, or mimic groundwater constituents. It was observed by Sheng et al. that the reductive removal of U(VI) by nZVI was enhanced by negatively charged Na^+^-saturated bentonite support, and this composite showed much higher removal efficiency for cationic U(VI) than either bare nZVI or NZVI supported on the positively charged Al-bentonite [127]. Na−bentonite facilitated the mass transfer of U(VI) from aqueous to iron surface, accelerated the reduction of U(VI) by nZVI, had a pH buffering effect, and could transfer the insoluble reduction products away from the iron surface, resulting in the improved stability and reusability of nZVI.

Zinc is often present as a pollutant in industrial wastewaters and environmental waters as a consequence of industrial activities and mining. These contaminated waters need to be cleaned up. Krzisnik et al. applied untreated, surface modified with octa(cholinium)-polyhedral oligomeric silsesquioxane (choline-POSS), and silica-fume supported nZVI for Zn(II) removal from water [32]. Untreated nZVI was the most efficient, and the Zn(II) removal was governed mainly by adsorption of Zn(II) onto precipitated iron oxides. Removal of strongly complexed Zn(II), namely Zn(II)–EDTA, was successful only at acidic pH, where Zn(II)–EDTA decomposed releasing Zn(II). Shi et al. used kaolin, bentonite, and zeolite supported nZVI for the simultaneous removal of Cu(II) and Zn(II) from aqueous solution [128]. nZVI/bentonite exhibited superior performance due to favorable dispersion and stabilization of nZVI on bentonite surface; this latter is possibly the result of the flexible lamellar structure of the clay. Kinetic studies confirmed the simultaneous removal of Cu(II) and Zn(II), where Cu(II) removal was based on reduction, while Zn(II) removal was adsorption. The efficiency of nZVI/bentonite in water purification was confirmed by cleaning industrial electroplating and dyeing wastewaters containing Pb(II), Cu(II), Zn(II), Ni(II), and Cr(VI) ions. Wang et al. compared the traditional lime method and nZVI treatment for their ability to remove Pb(II) and Zn(II) from wastewater [129]. nZVI treatment had several advantages as a lower impurity level could be achieved and it was able to generate large and consolidated solids that were easier to separate by gravitational separation. A field study was conducted to remove Pb(II) and Zn(II) from wastewater generated by a smelting plant, using tons of lime and kilos of nZVI. The pilot experiment suggested that nZVI could be applied as an advanced treatment process to remove the residual metal ions in the effluent of lime treatment. 

**Figure 9:**
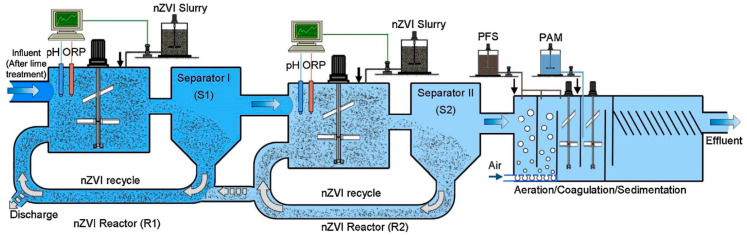
Schematic of a wastewater treatment process using nZVI. Reproduced with permission from [130]. Copyright Elsevier Inc., 2017.

Wastewater, depending on the source, contains multiple contaminants, therefore wastewater purification has to consider simultaneous removal of different ions, as well as system reliability and quick separation of reaction products. Li et al. demonstrated that nZVI is able to remove different heavy metals and arsenic simultaneously, thus nZVI is an ideal reagent for wastewater treatment [130]. Field experiments were performed at the Jiangxi Copper Company. The constructed treatment facility is shown in Figure 9. A very acidic wet hydrometallurgical processes wastewater with very high Cu(II) and As(V) concentration and a mixture of heavy metal cations (Au, Cu, Co, Cr, Ni, Tl, Zn, and Pb) and oxyanions (e.g., As, Se, and Sb) were purified. The purification process removed >90% arsenic and nearly all copper, and reduced the concentration of other elements to well below 0.1 mg/L. Iron and the mentioned eleven impurities were identified in the spent nZVI; their relative abundance followed a similar order as in the influent wastewater. nZVI served as solid seeds to facilitate product separation, and the removal mechanisms of nZVI included reduction, sorption, and precipitation depending on the contaminant.

**Table 6:** Heavy metal ion removal from water ^1^.

nZVI Cap/Support	Pollutant	pH	Adsorption Capacity (mg/g)	Ref.
Graphene-silica	As(III)	6.0–8.0	45.57	[101]
Graphene-silica	As(V)	4.0	45.12	[101]
Montmorillonite	As(III)	7.0	59.9	[103]
Montmorillonite	As(V)	7.0	45.5	[103]
Bare	As(III)	7	102	[104]
Bare	As(V)	7	118	[104]
Bare	As(V)	7	26.36	[106]
Zeolite	As(V)	7	47.30	[106]
Fe-oxide	As(V)	6–8	245	[130]
FeOOH	Au(III)	neutral	25	[107]
Biochar	Cd(II)	6	22.37	[108]
rGO	Cd(II)	5	425.72	[49]
kaolinite	Co(II)	n.a.	25	[110]
rGO	Co(II)	4–9	131.58	[111]
kaolinite	Cu(II)	n.a.	140	[110]
Bare	Cu(II)	n.a.	250	[116]
MWCNT-PAA/PVA	Cu(II)	4.5–5	107.8	[118]
alumina	Cu(II)	3–11	95.3	[117]
Fe-oxide	Cu(II)	6–8	226	[130]
Biochar	Cr(VI)	5	26.63	[112]
Lignin/Al-bentonite	Cr(VI)	5.6	46.2	[113]
CMC	Cr(VI)	7	3.33	[114]
MWCNT	Cr(VI)	7	2.71	[114]
pumice	Cr(VI)	n.a.	23.6	[27]
Carbon nanofiber	Cr(VI)	4	n.a.	[115]
pumice	Hg(II)	n.a.	25.6	[27]
FeOOH	Ni(II)	neutral	130	[120]
Bare	Pb(II)	6	807.23	[22]
Bare	Pb(II)	neutral	1718.4	[121]
Mg(OH)_2_	Pb(II)	neutral	1986.6	[121]
zeolite	Pb(II)	4	806	[122]
CMC	Se(IV/VI)	7	2.26	[114]
MWCNT	Se(IV/VI)	7	2.52	[114]
Fe-oxide/PVDF/PAA	Se(IV/VI)	4.5	n.a.	[124]
Bare	Se(IV)	neutral	n.a.	[9]
Bare	U(VI)	5	8173	[126]
rGO	U(VI)	5	4174	[126]
Na-bentonite	U(VI)	<7	120	[127]
Bare	Zn(II)	5–7	n.a.	[32]
bentonite	Zn(II)/Cu(II)	3.9	n.a.	[128]

^1^ n.a. = not available; Bare = NPs where a shell is not produced intentionally (contains a self-developed iron oxide layer); PVDF = polyvinylidene fluoride; PAA = polyacrylicacid; PVA = polyvinyl alcohol.

#### 4.4.2. Removal of Heavy Metal Contaminants from Groundwater and Soil 

Industrial activity often results in polluted soil if proper technology is not used, and consequences usually become more obvious after the closure of industrial plants. Baraganno et al. investigated the possible remediation of arsenic polluted soil of a former fertilizer plant using commercial nZVI [131] (Table 7). The soil was a sandy loam containing 30-times more As than the maximum permitted level (>96% was arsenate and arsenite was below 4%). nZVI was effective in immobilizing As in the polluted soil, the reduction of As(V) was minimal, and the main immobilization process was the adsorption of arsenate onto iron oxides in the shell surrounding the Fe^0^ core of NPs through inner-sphere surface complexation. The application of nZVI did not affect the pH or the electrical conductivity of the soil, increased slightly the availability of Fe, and reduced significantly the phytotoxicity.

Cadmium is a pollutant of many river sediments especially close to relevant industrial and mining sites, therefore development of remediation technology for the effective immobilization of Cd is important. Xue et al. investigated the influence of nZVI and rhamnolipid (RL) biosurfactant modified nZVI on Cd mobility in Cd-bearing sediments collected from the Xiangjiang River [31]. NPs were synthesized using the liquid phase borohydride route. Compared to nZVI, nZVI/RL was more effective in transforming labile Cd to stable fraction, likely by adsorption or complexation processes. The nZVI/RL treatment changed the bacterial community structure in the sediment and increased the relative abundance of Fe(III)-reducing bacteria.

Sediment exhibits a high tendency of binding and thus reserving toxic and persistent compounds of predominantly anthropogenic origin [132]. Pilipovic et al. synthesized bentonite, caolinite, and CMC supported nZVI for the stabilization of nickel, lead and zinc in sediment taken from the most ecologically endangered sections of Nadela (Serbia) watercourse [132]. The soil was injected with nZVI NPs and mixed for homogeneous distribution. All three supported nZVI composites were effective in reducing mobile Ni(II), Pb(II), and Zn(II) contents, but CMC-nZVI exhibited the best performance, with over 50% removal after four weeks, possibly due to longer ageing effect and better transport in porous media.

Nemecek et al. applied a combination of nZVI and whey to reduce and immobilize Cr(VI) in groundwater at the Kortan site in Hradek nad Nisou, Czech Republic [133]. The pilot test consisted of consecutive nine months abiotic and fifty day biotic phases, where suspension of commercial nZVI in water and whey, respectively, were injected into the ground through injection wells. The abiotic phase resulted in a rapid decrease in Cr(VI) concentrations by nZVI, and facilitated the subsequent use of the cheaper biological method. The effect of biotic reduction was observed even after ten months in a monitoring well located 22 m far from the injection wells. The same Cr(VI) contaminated site, formerly used for Cr(III) salt production from potassium dichromate for leather tanning, was used by Nemecek et al. to study the Cr(VI) removal efficiency of commercial nZVI [134]. Upon nZVI treatment, both Cr(VI) and total Cr concentrations decreased rapidly in the groundwater without any substantial effect on its chemical properties. A combination of nZVI and whey was also applied by Nemecek et al. to remediate a polluted site contaminated with chlorinated ethenes, mainly trichloroethene and cis-dichloroethene, and Cr(VI) originating from historical degreasing and Cr coating activities [135]. The aquifer was developed in Quaternary sandy gravels. nZVI was efficient for the complete removal of Cr(VI) from the groundwater, but interestingly the chlorinated ethane concentration hardly decreased in the presence of Cr(VI). nZVI, in general (see above), is an efficient dehalogenating agent for chlorinated organic compounds. The inefficiency of nZVI to reduce chlorinated ethenes in this case was due to the thermodynamically more favored reduction of Cr(VI). Consecutive application of whey resulted in effective removal of chlorinated ethenes.

### 4.5. Removal of Ammonium, Bromate, Nitrate, and Perchlorate Ions from Water 

Nitrate appears extensively in industrial wastewater, ground water, and livestock wastewater as a contaminant. In high concentration it causes health problems, therefore a limit of 50 mg/L in drinking water is set by the World Health Organization (WHO) [136]. nZVI is an effective nitrate reducing material. Chen et al. synthesized cetylpyridinium chloride (CPC) stabilized nZVI by combining electrochemical and ultrasonic methods [46], and used nZVI for nitrate reduction in water (Table 8). NPs were gradually deactivated during the process due to iron oxide film formation. Nitrate was reduced to ammonium ions and nitrogen gas depending on pH. Hwang et al. synthesized Mg-aminoclay stabilized nZVI particles for nitrate decontamination of water [28]. The higher stability of coated compared to uncoated nZVI could be explained by the increased electrostatic repulsion between positively charged Mg-aminoclay coatings. Coated NPs exhibited higher reactivity to nitrate ions and converted nitrate partially to ammonium ions. Ryu et al. tested the effectiveness of freshly synthesized, dried, and dried sonicated nZVI in nitrate reduction [137]. All these different nZVIs could effectively reduce highly concentrated nitrate without requiring pH control, but the freshly prepared NPs were the most reactive. Aggregate size strongly affected the reduction rate. Ammonium was the final, and nitrite the byproduct of the reaction, and nZVI changed into magnetite. Diao et al. synthesized nZVI from acid mine drainage using sodium borohydride, and applied the prepared nZVI particles for nitrate removal from water under ultrasonic irradiation [73]. nZVI, containing also Cu and Al from the drainage, effectively reduced nitrate, and ultrasonic irradiation enhanced the reaction rate through fluidizing and depassivating nZVI. The removal efficiency of nitrate was significantly dependent on solution pH and decreased as pH values increased from 2 to 8. Coexisting cations in the prepared solution had a negligible effect on nitrate removal, however, anions, especially phosphate and hydrogencarbonate, inhibited the nitrate removal possibly due to adsorption and coprecipitation on the catalyst surface. Nitrate ions were effectively transformed into ammonium ions. Vilardi et al. studied the intensification of nZVI-induced denitrification of water by means of a spinning disk reactor [138]. This intensified equipment exhibited better performance in nitrate removal from water, namely larger removal efficiency at a shorter contact time, than classical batch reactor.

Liu et al. developed a fly ash-derived zeolite composite incorporating nZVI for ammonium ion removal from water, by mixing a zeolite precursor with commercial nZVI particles and crystallizing the black gel in a Teflon-lined stainless steel autoclave [139]. nZVI was incorporated into the framework of zeolite, some occupied micropores, and some was highly dispersed on the surface of the zeolite. The ammonium ion removal from water was explained by the adsorption of ammonium ions on iron hydroxide.

Perchlorate is now a widespread contaminant in groundwater and surface water [140]. Perchlorate anion has an unusual stability in aqueous solution and in general it is non-reactive and does not form complexes. It is therefore difficult to remediate perchlorate contamination from water. Cao et al. synthesized nZVI with an average diameter of 57 nm, and successfully applied nZVI NPs to reduce perchlorate to chloride in water [141]. The reduction reaction was observed to be slow at room temperature due to the relatively large activation energy for the perchlorate–iron reaction. The high surface area and reactivity was essential for this reaction as no reaction was observed with microscale iron powder. nZVI reduced also chlorate, chlorite, and hypochlorite ions, with much faster reaction rate. Xiong et al. also observed that nZVI decomposed perchlorate to chloride without the accumulation of any intermediate products in a relatively short time at moderately elevated temperatures both in water or ion-exchange brine [140]. Starch and CMC-stabilized nZVI were more efficient than unsupported nZVI.

Bromate is a highly persistent and carcinogenic byproduct of the oxidative disinfection of drinking water containing bromide. Wang et al. investigated the potential application of nZVI for bromate reduction in drinking water systems [142]. nZVI was found to be effective in bromate removal not only from drinking water but river water samples. nZVI was more reactive than microsized ZVI [142]. 

## 5. Toxicity of nZVI 

Iron compounds are widely distributed in nature and iron is detected almost in all natural waters, therefore, iron is regarded as a green material. nZVI, however, is very reactive and this reactivity raises concerns about its potential negative effects on the environment [4,40,143,144]. Various kinds of microorganisms such as iron reducers, sulfate reducers, and methanogens, etc. are present under different conditions in the subsurface. In general, nZVI is a strong reducing agent thus it can create a reductive atmosphere and anoxia, e.g., for microorganism, it generates reactive radical species at the presence of oxygen thus may create oxidative stress, it may increase iron ion content in the environment and decrease the concentration of many vital components due to the reaction, which may favor or disfavor certain microorganism populations. The reduction of a certain pollutant may result in the temporary formation of a more toxic material than the target compound itself (e.g., vinyl chloride formation from chlorinated hydrocarbons). In addition, the effect of support or capping material of nZVI and their fate in the environment have to be also taken into account. Environmental effects of different forms of nZVI cannot directly be compared. It also has to be considered that the microflora in polluted soil is already altered compared to uncontaminated soil due to the toxic effects of contaminants. The change in microbial composition is not necessarily negative. Consequently, there are many important factors that have to be considered before nZVI is applied on a wide scale in the environment. Pilot field tests are therefore very important. There is an increasing number of laboratory and field works addressing the toxicity of nZVI.

Nemecek et al. studied the effects of the application of commercial nZVI on the indigenous bacteria and groundwater toxicity after the remediation a Cr(VI) contaminated site formerly used for leather tanning [134] (Table 9). The luminescent bacterium *Vibrio fischeri* was used for the toxicity test, and the ecotoxicological test did not indicate any negative changes in the toxicity of the groundwater. Sulfate-reducing bacteria was not detected in the groundwater before treatment, and anaerobes and facultative anaerobes were in very low amounts. nZVI injection in soil did not negatively affect the viability of these bacterial populations in the groundwater. Microbial cultivation tests of psychrophilic bacteria did not show any clear changes (this bacterial population is sensitive to Cr(VI)). In soil samples, however, the application of nZVI stimulated the growth of G+ bacteria and there was a clear correlation between the concentration of iron and bacterial number.

Bacterial communities are sensitive to environmental changes. Xue et al. investigated the effect of nZVI treatment of river sediments on the bacterial community structure [31]. Proteobacteria, Acidobacteria, Bacteroidetes, and Gemmatimonadetes were the most four predominant bacterial phyla, and the community contained also Firmicutes, Chloroflexi, Planctomycetes, Actinobacteria, and Verrucomicrobia phyla. nZVI treatment particularly positively increased the abundance of Proteobacteria and Firmicutes, including the genus *Geobacter* and *Bacillus*, which are responsible for the reduction of Fe(III) minerals.

Sun et al. studied and compared the toxicity of bare nZVI, mesoporous silica SBA-15 supported nZVI, and nZVI confined in the mesochannels of SBA-15 on *Escherichia coli* [145]. The composite with nZVI confined in the mesochannels had minimal toxicity to *Escherichia coli*, but both the bare and the surface supported NPs had a strong bactericidal effect. The minimal toxicity of nZVI confined in the mesochannels of SBA-15 might be explained by the lack of direct contact between nZVI and bacteria cell due to the electrostatic hindrance resulting from the silica host.

nZVI is able to generate reactive oxygen species at the presence of oxygen, which can cause oxidative stress for microorganisms. Semerad et al. developed a sensitive and simple method for the determination of aldehydes in microbial cultures, which are typical markers of oxidative stress, and tested the effect of nZVI on representatives of bacteria (*Bacillus cereus* and *Serratia marcescens*), yeast (*Saccharomyces cerevisiae*), and algae (*Desmodesmus subspicatus*) [146]. In the laboratory experiments, the respective microbial suspension was treated with commercial nZVI NP suspension. nZVI was found to cause oxidative damage to both proteins and lipids. All microorganisms produced markers of oxidative stress. The yeast strain was found to be the most sensitive to the oxidative stress.

Khuntia et al. tested the ecotoxic effect of pectin-capped nZVI on Collembola (*Folsomia candida*), a soil arthropod, and Ostracods (*Heterocypris incongruens*), a sediment dweller, in spiked soil [30]. Quite severe negative effects of nZVIs on the mortality and growth of both Collembola and Ostracods were observed. The study indicated that the Ostracod mortality might be indirect and is the result of a state of anoxia created by the consumption of oxygen by nZVI NPs.

Adeleye et al. investigated the properties, aggregation, and transformation of sulfide/silica-modified nZVI in freshwater algal media using *Chlamydomonas reinhardtii* [147]. Steric stabilization of NPs by algal organic matter was observed, which led to a decrease in attachment efficiency of NPs. High concentrations of nZVI caused a lag in algal growth. The growth rate was initially lower compared to controls in the exponential phase, but eventually grew to the same steady state population size as control cultures. In contrast, 11-day old cultures did not exhibit any obvious negative effects and were still growing after 36-days when the control cultures started to decline probably due to cell senescence.

Applying nZVI for soil remediation, plants are possibly also exposed to nZVI. Liu et al. investigated the potential phytotoxicity of nZVI NPs to *Arabidopsis thaliana* plant. Fe nanoparticles up to 100 mg L^−1^ did not show toxic effects to the growth of *Arabidopsis thaliana* [87]. Kim et al. investigated the physiological effects of nZVI in rhizosphere on edible crop, *Medicago sativa*, grown in soil mixed with nZVI [148]. No physiological phytotoxicity was observed in plants, although nZVI-mediated OH∙ radicals induced cell wall loosening was detected. nZVI-treated plants, compared to non-treated, exhibited higher chlorophyll concentration in 20-day-old seedlings, slightly lower carbohydrate and lignin contents, and translocation of nZVI into plant tissues.

## 6. Conclusions and Outlook 

nZVI possess several advantages over bulk or microscale iron materials due to its small size, which manifests in higher surface area and reactivity, higher adsorption capacity, and higher mobility, especially when this latter property is increased by modifiers. Past and present anthropogenic pollutions possess a threat to the environment and human health, and are now the focus of global attention. nZVI is reactive to many organic and inorganic toxic pollutants in the environment, and since iron is widely distributed in nature, nZVI has become one of the most important nanomaterials for environmental remediation and wastewater purification. One of the advantages of the application of nZVI is that these NPs can be injected into the immediate vicinity of contaminant sources. Replacing toxic materials with iron in the environment seems to be a good alternative. Although nZVI has opened a new door for treating contaminated groundwater and soil, as well as industrial and municipal wastewaters, it is also accompanied by new potential risks if introduced into the environment on a large scale. Despite of favorable properties of nZVI, there are limitations for its applications rising from low mobility of NPs in various media, high reactivity with oxygen and water, and tendency to aggregate. Currently there is an intensive research to stabilize and modify these NPs to increase transportability, mobility in water and soil, adsorption properties, and reactivity. A wide range of support and capping materials have been used to this end. There is a synergistic effect between the iron core and modifiers, and a fine balance between reduced reactivity and increased stability, mobility, and adsorption properties. There is a quest for producing novel modified nZVI NPs fitting better for specific application requirements, and this along with field and environmental impact studies will increase in the future. An important aspect of future researches will be to answer the challenge to scaling-up production to produce modified nZVI on an economical and large scale. The currently widely used borohydride synthetic route is limited due to the relatively high costs of the required chemicals and toxicity of the reagent. Support materials are expected also to be in the focus of future researches as requirements are more critical toward biodegradability, environmental friendliness, and natural and renewable sources. There are advantages and disadvantages of the application of nZVI in water purification and environmental remediation, however, the relatively low production cost, low toxicity, high reactivity, and high surface area highlight this nanomaterial as one of the most important materials in purification and remediation processes. There are concerns about the direct environmental application of nZVI concerning its effects on microorganisms and plants, however, this is a complex question; not only because both positive and negative effects were observed, but also the long term benefit of purifying a toxic water or soil is of the main importance. It must be noted that long term impacts of nZVI on the environment cannot adequately predicted from acute toxicity studies, because toxic effects of NPs change with their transformation in the environment. Currently, long term field studies for the environmental impact of nZVI are very scarce. In addition, biological effects are not directly relevant to the on-site industrial effluent purifications. A bright future is expected for the application of nZVI, and both laboratory and field studies are expected to increase in the future to improve properties and to understand reaction mechanisms and long term biological effects.

## Figures and Tables

**Table 1:** Catalytic application of nZVI ^1^.

Support/Cap	Precursor	Reagent	Product	Reference
rGO	Ar-NO_2_^2^	N_2_H_4_	Ar-NH_2_	[47]
no support	*p*-nitrophenol	NaBH_4_	*p*-aminophenol	[57]
PEG, CMC, or PVP ^3^	Ketones ^4^	NaNH_4_	alcohols	[23]
Octanoic acid, bis-2-ethylhexylamine	CH_4_	—	SWCNT	[25]

^1^ n.a. = not available; ^2^ Ar = phenyl, o-chloro-phenyl, m-bromo-phenyl, p-(chloro, cyano, methoxy, methyl, amino, trifluoromethyl, aminocarbonyl)-phenyl, 2-pyridyl; ^3^ PVP = polyvinylpyrrolidone, PEG= polyethylene glycol, CMC= carboxymethyl cellulose; ^4^ Aromatic ketones: phenyl-methyl, *p*- and *o*-nitro-phenyl-methyl, *p*- and *o*-methoxy-phenyl-methyl, *p*-chloro-phenyl-methyl, *p*-bromo-phenyl-methyl, *p*-methyl-phenyl-methyl, *i*-butyl-phenyl-methyl, phenyl-chloromethyl.

**Table 2:** Application of nZVI for organic compound removal from water and soil ^1^.

nZVI Cap/Support	Pollutant	pH, Reagent	Adsorption Capacity (mg/g) or dec. Product	Ref.
From water				
Silica/PDA ^2^	Anthracene	3–11	0.367	[68]
Silica/PDA	Phenanthrene	3–11	0.185	[68]
Al(OH)_3_	4-nitrophenol	7.3	4-aminophenol	[26]
Carbon	Phenol	4–5, H_2_O_2_	n.a.	[50]
Bare	Phenol	4, H_2_O_2_	CO_2_, H_2_O	[45]
Bentonite	Phenol, Cr(VI)	5, S_2_O_8_^2−^	Formic acid	[69]
Diatomite	Bisphenol A	5.75, H_2_O_2_	CO_2_, H_2_O	[70]
Bare	17α-ethinylestradiol	3, 5, 7, O_2_	C_20_H_28_O_2_	[59]
polyphenols	Amoxicillin	3, H_2_O_2_	CO_2_, H_2_O	[41]
PVP	Metronidazole	5.6	C_6_H_11_N_3_O	[71]
PEG	Amoxicillin	6.6	AMX penicilloic acid	[72]
PEG	Ampicillin	6.6	AMP penicilloic acid	[72]
Bare	Norfloxacin	4, air	CO_2_, H_2_O	[73]
PVP	Tetracycline	6.5	C_19_H_26_O	[74]
Sepiolite	Metoprolol	3, H_2_O_2_	n.a.	[75]
From spiked soil				
Polyphenols	Amoxicillin	2.6–3.4, H_2_O_2_	CO_2_, H_2_O	[41]
Bare	Malathion	7.6	ODP	[76]

^1^ n.a. = not available; Bare = NPs where a shell is not produced intentionally (contains a self-developed iron oxide layer); see Table 3 and Table 4 for organic dyes and antibiotics; ^2^ PDA = polydopamine; PVP = polyvinylpyrrolidone; ODP= *O*-dimethyl phosphorodithioic derivative.

**Table 5:** Removal of halogenated organic compounds from soil ^1^.

nZVI Cap/Support	Pollutant	Soil	pH, Product	Ref.
Bare	Vinyl chloride	Ground water	6–7, methane, ethene	[95]
CMC	PCE and TCE	Sediment	Ethene	[96]
Emulsion	TCE	Soil	Ethene ^2^	[97]
Emulsion	TCE	Soil	Ethene ^2^	[98]
Pectin	DDT	Spiked soil	5.9	[30]
MEG	PCE	Sand layers	Ethene	[14]
Bare	PCE	Soil	Ethene ^2^	[99]

^1^ Bare = NPs where a shell is not produced intentionally (contains a self-developed iron oxide layer); DDT = dichlorodiphenyltrichloroethane; MEG = mono ethylene glycol; PCE = tetrachloroethene or perchloroethylene; TCE = trichloroethene; ^2^ *cis*-dichloroethene and vinyl chloride were also detected in the groundwater.

**Table 7:** Removal of heavy metal and metalloid ions from soil ^1^.

nZVI Cap/Support	Pollutant	Soil	pH	Ref.
Bare	As(V)	Sandy loam	n.a.	[131]
Rhamnolipid	Cd(II)	River sediment	7.71	[31]
CMC	Ni(II), Pb(II), Zn(II)	River sediment	n.a.	[132]
Bentonite	Ni(II), Pb(II), Zn(II)	River sediment	n.a.	[132]
Caolinite	Ni(II), Pb(II), Zn(II)	River sediment	n.a.	[132]
Bare	Cr(VI)	Groundwater	5.4	[133]
Bare	Cr(VI)	Groundwater	5.4	[134]
Bare	Cr(VI) ^2^	Groundwater	n.a.	[135]

^1^ n.a. = not available; Bare = NPs where a shell is not produced intentionally (contains a self-developed iron oxide layer); ^2^ soil contained also chlorinated ethenes.

**Table 8:** Inorganic oxoanion and ammonium cation removal from water ^1^.

nZVI Cap/Support	Pollutant	pH, Reagent	Adsorption Capacity (mg/g) or Product	Ref.
CPC	NO_3_^−^	4–7	NH_4_^+^, N_2_	[46]
Mg-aminoclay	NO_3_^−^	8.8	NH_4_^+^	[28]
Bare	NO_3_^−^	n.a.	NH_4_^+^, NO_2_^−^	[137]
Bare	NO_3_^−^	4	NH_4_^+^	[73]
CMC	NO_3_^−^	n.a.	n.a.	[138]
Zeolite	NH_4_^+^	8	62.82	[139]
Starch	ClO_4_^−^	7–7.4	Cl^−^	[140]
CMC	ClO_4_^−^	7–7.4	Cl^−^	[140]
Bare	ClO_4_^−^	6	Cl^−^	[141]
Bare	ClO_3_^−^	6	Cl^−^	[141]
Bare	ClO_2_^−^	6	Cl^−^	[141]
Bare	ClO^−^	6	Cl^−^	[141]
Fe_3_O_4_/γ-Fe_2_O_3_	BrO_3_^−^	3‒7	Br^−^	[142]

^1^ n.a. = not available; Bare = NPs where a shell is not produced intentionally (contains a self-developed iron oxide layer); CPC = cetylpyridinium chloride; CMC = carboxymethyl cellulose.

**Table 9:** Toxicity of nZVI ^1^.

nZVI Cap/Support	Organism	Effect	Cause of Toxicity	Ref.
Bare	*Vibrio fischeri*	No	—	[134]
Bare	G+ bacteria	Positive	—	[134]
Rhamnolipid	*Proteobacteria* and *Firmicutes*	Positive	—	[31]
Pectin	Collembola (*Folsomia candida*)	Negative	n.a.	[30]
Pectin	Ostracods (*H. incongruens*)	Negative	Anoxia	[30]
Bare	*Escherichia coli*	Negative	Reduction	[145]
SBA-15 ^2^	*Escherichia coli*	Minimal	—	[145]
Bare	*Bacillus cereus*	Negative	Oxidative stress	[146]
Bare	*Serratia marcescens*	Negative	Oxidative stress	[146]
Bare	*Saccharomyces cerevisiae*	Negative	Oxidative stress	[146]
Bare	*Desmodesmus subspicatus*	Negative	Oxidative stress	[146]
Sulfide/silica	*Chlamydomonas reinhardtii*	Lag	—	[147]
Bare	*Arabidopsis thaliana*	No toxic	—	[87]
Bare	*Medicago sativa*	No toxic	—	[148]

^1^ n.a. = not available; Bare = NPs where a shell is not produced intentionally (contains a self-developed iron oxide layer); ^2^ nZVI confined in the mesochannels of mesoporous silica SBA-15.
